# Occupational exposure to hexavalent chromium: a systematic review of environmental monitoring methods and analytical advances

**DOI:** 10.1093/annweh/wxag047

**Published:** 2026-06-22

**Authors:** Andrea Spinazzè, Francesca Borghi, Carolina Zellino, Veronica Prina, Andrea Cattaneo, Sandro Recchia, Carlo Dossi, Salvatore Della Notte, Veruscka Leso, Ivo Iavicoli, Domenico Maria Cavallo

**Affiliations:** Department of Science and High Technology, University of Insubria, Via Valleggio 11, Como, CO 22100, Italy; Department of Science and High Technology, University of Insubria, Via Valleggio 11, Como, CO 22100, Italy; Department of Medical and Surgical Sciences, University of Bologna, via P. Palagi 9, Bologna 40138, Italy; Department of Science and High Technology, University of Insubria, Via Valleggio 11, Como, CO 22100, Italy; Department of Science and High Technology, University of Insubria, Via Valleggio 11, Como, CO 22100, Italy; Department of Science and High Technology, University of Insubria, Via Valleggio 11, Como, CO 22100, Italy; Department of Science and High Technology, University of Insubria, Via Valleggio 11, Como, CO 22100, Italy; Department of Theoretical and Applied Sciences, University of Insubria, Via O. Rossi, 9, Varese 21100, Italy; Department of Public Health, Section of Occupational Medicine, University of Naples Federico II, Via S. Pansini 5, Naples 80131, Italy; Department of Public Health, Section of Occupational Medicine, University of Naples Federico II, Via S. Pansini 5, Naples 80131, Italy; Dipartimento di Sicurezza e Bioetica, Catholic University of Sacred Heart, Largo Francesco Vito 1, Rome 00168, Italy; Fondazione Policlinico Universitario A. Gemelli, IRCCS, Largo Agostino Gemelli 8, Rome 00168, Italy; Department of Science and High Technology, University of Insubria, Via Valleggio 11, Como, CO 22100, Italy

**Keywords:** chromate, Cr(VI) speciation, inhalable fraction, alkaline extraction, diphenylcarbazide, ISO 16740, NIOSH 7600, welding fumes, ultrafine particles, exposure assessment, biomonitoring, risk assessment

## Abstract

**Background:**

Hexavalent chromium (Cr(VI)) is a well-established occupational carcinogen, widely utilized in industrial processes such as electroplating, surface treatment, and ferrochromium production. It is also generated as a by-product of welding activities. Accurate monitoring of occupational exposure to Cr(VI) is essential for protecting workers' health. This review aims to critically assess the main challenges associated with existing environmental monitoring techniques for Cr(VI) in welding operations and to propose practical strategies to address these limitations.

**Methods:**

A systematic literature review was conducted by consulting 3 scientific databases (Scopus, Web of Science, and PubMed). Studies assessing occupational exposure to Cr(VI) published since 2014, were included and analyzed in terms of methods, dosimetric parameters measures, and possible alternative approaches for Cr(VI) characterization.

**Results:**

The reviewed studies employed diverse environmental monitoring strategies for occupational Cr(VI) exposure assessment, with substantial heterogeneity in sampling conventions, analytical workflows, and speciation capability. Some studies additionally reported biological measurements and/or used exposure modeling as supportive approaches; however, these were not systematically reviewed as primary endpoints. Findings confirm that occupational exposure to Cr(VI) remains a concern in multiple industries, with exposure levels varying according to tasks, process characteristics, and preventive measures. However, major challenges persist. Key issues include the difficulty of distinguishing Cr species, the instability of Cr(VI) during sampling and analysis, and the scarcity of reliable speciation data. Furthermore, particle size distribution—especially the role of ultrafine particles—remains poorly characterized despite its toxicological importance. Innovative tools, including advanced analytical methods and modeling approaches, show promise but require further validation.

**Conclusions:**

To fill research gaps and improve risk assessment, future studies should (i) accurately differentiate between chemical species of metals; (ii) adopt methods capable of measuring particle size distribution, with focus on ultrafine fractions; and (iii) systematically collect contextual data on Personal Protective Equipment use and work activities.

What is important about this paper This systematic review synthesizes evidence on how occupational exposure to hexavalent chromium is measured across key industries. It shows that integrated approaches combining air monitoring with contextual determinants are increasingly reported; some studies also included biomonitoring as contextual support. However, chemical instability and limited speciation remain major sources of uncertainty. By mapping sampling fractions and analytical methods at the study level, this review provides practical guidance for selecting fit-for-purpose protocols.

Highlights

- The study is a systematic review of the literature on occupational exposure to Cr(VI).
- The review analyzed 79 studies, published since 2014, conducted in various occupational settings.
- The review highlights integrated exposure assessment, centered on airborne Cr(VI) monitoring and supported by contextual information and complementary indicators.
- The study identified several critical challenges, notably the difficulty in differentiating between various chemical Cr species. Future research should focus on developing innovative technologies for monitoring nanoparticles.

## Introduction

Chromium (Cr) is a heavy metal, widely employed for industrial processes that can be harmful for humans, causing skin allergies, asthma, pneumonitis, pharyngitis, hepatic diseases, and increasing the risk of lung cancer in exposed subjects (IARC Working Group on the Evaluation of Carcinogenic Risks to Humans. and International Agency for Research on Cancer 1990; Dayan and Paine 2001; Heederik et al. 2016; Deng et al. 2019; Alvarez et al. 2021). Chromium speciation is an extensively studied topic owing to the striking difference in toxicity of its 2 most stable oxidation states, the trivalent (Cr(III)) and hexavalent (Cr(VI)) forms (Dayan and Paine 2001; Heederik et al. 2016; Alvarez et al. 2021). The former is considered an essential nutrient for living organisms playing a crucial role in the metabolism of glucose and lipids and is characterized by a low toxicity profile (Heederik et al. 2016). Oppositely, Cr(VI) is classified as Group 1 (carcinogenic to humans) by the International Agency for Research on Cancer (IARC) (IARC Working Group on the Evaluation of Carcinogenic Risks to Humans. and International Agency for Research on Cancer 1990), and chronic exposure to Cr(VI) can cause severe damage to human health (Gatto et al. 2010; Gibb et al. 2015; Yatera et al. 2018; Deng et al. 2019; Alvarez et al. 2021; Hessel et al. 2021; Wise et al. 2022). The potential risks associated with Cr exposure are critically relevant considering that workers in various industrial sectors can be exposed through inhalation and dermal contact: in plating/surface-treatment operations and chromate-containing coatings, Cr(VI) may be directly used, whereas in high-temperature metalworking (eg stainless-steel welding/cutting and thermal spraying) Cr(VI) can be generated in situ; in the leather industry, chromium is predominantly present as Cr(III), with Cr(VI) potentially arising under specific oxidation conditions. Occupational exposure to (Cr(VI)) falls under the scope of occupational safety and health legislation governing exposure to carcinogenic and mutagenic substances in the workplace (European Parliament 2006; European Parliament and Council 2022). Specifically, Cr(VI) exposure is regulated through OSH provisions addressing carcinogens and mutagens (European Parliament 2006; European Parliament and Council 2022).

Accurate measurement of total Cr(VI), including both soluble and insoluble fractions, is essential. Occupational exposure is typically assessed by monitoring airborne particles and comparing Cr(VI) levels to occupational exposure limit values (OELVs) (CEN—European Committee for Standardization 2019). The current EU directive (European Parliament 2017) sets the OELV at 5 μg/m^3^. Some Countries have adopted even stricter limits (Santonen et al. 2022). Currently, official methods for measuring total Cr(VI) in airborne particulate matter (PM) include the ISO 16740:2005 standard, NIOSH 7600-7605, and OSHA ID-215 (Boiano et al. 2000). These methods involve collecting PM on polyvinylchloride (PVC) filters, detecting Cr(VI) using a 1,5-diphenylcarbazide (DPC) colorimetric method, and using alkaline extractants at 95 °C (Boiano et al. 2000). However, these methods can cause issues with the interconversion of Cr(III) to Cr(VI) during hot alkaline extraction, potentially leading to overestimation of Cr(VI) (Unceta et al. 2010). This is particularly problematic in environments with prominent levels of Cr(III). Accurate environmental monitoring must consider method sensitivity, Cr(VI)/Cr(III) stability, and proper sampling and preservation of collected particles.

This study aims to critically examine the currently available procedures for assessing occupational exposure to Cr(VI) through environmental monitoring. The objective is to identify existing challenges and limitations and to propose practical solutions and opportunities—particularly through the adoption of innovative analytical methods—to enhance the overall chemical risk assessment process. Within this framework, a comprehensive state-of-the-art review of current environmental monitoring techniques for Cr(VI) exposure in workplace settings has been conducted. The findings will serve as a foundation for future research aimed at accurately characterizing occupational exposure risks and deepening the understanding of Cr(VI)-related hazards. Ultimately, this work seeks to inform and support the development of more effective risk management policies by highlighting critical issues for further investigation and identifying key factors to consider when evaluating potential risks associated with Cr(VI) exposure in occupational environments.

## Materials and methods

The results from 3 different scientific databases (Scopus, Web of Science, and PubMed) were considered in this systematic review. For each database, a list of keywords was arranged in a search query, as reported in Table S1. A total of 1,394 papers were found (771, 226, and 397 papers in Scopus, Web of Science, and PubMed, respectively). Duplicates (*n* = 574) were removed. The articles were then selected based on consistency with the aims of this study. Selected studies were those concerning occupational exposure to Cr(VI) published from 2014, that (a) presenting data from environmental monitoring and/or (b) presenting new methods of measurement and analysis of exposure to Cr(VI), and/or (c) discussing limitations or problems of current methodological approaches, and/or (d) suggesting possible innovative parameters or alternative approaches to measurement with classical methods. Speciation was not used as an exclusion criterion, as one of the objectives of this review was to explicitly quantify the extent of non-speciated monitoring in current practice. The articles were screened by (i) title (666 papers removed) and (ii) abstract (67 papers removed). The remaining papers (full-text reading) were then selected based on the following inclusion and exclusion criteria chosen a priori. Only scientific papers written in English were considered in this review, excluding conference papers and review articles. Only the articles that meet the above-mentioned inclusion criteria and aims of the review were examined. After that, 79 papers were finally included in this review (Table S2). The selection and the screening process of articles were conducted separately by different authors (A.S., F.B., C.Z., and V.P.), to reduce operator-related errors. The selection of the papers to be reviewed was performed following the PRISMA (Preferred Reporting Items for Systematic reviews and Meta-Analyses) criteria guidelines (Moher et al. 2009; Page et al. 2021).

**Figure 1:**
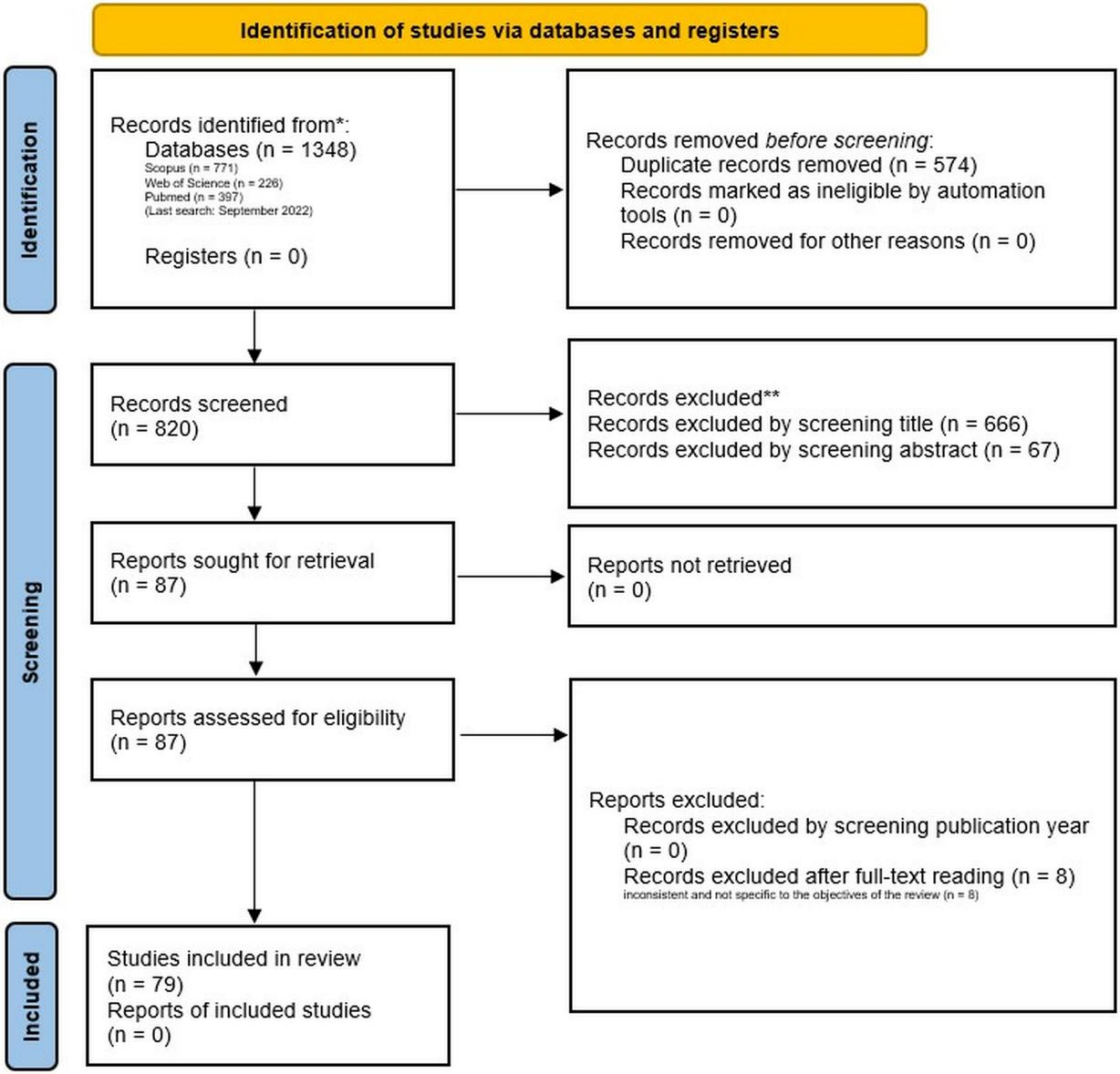
Flowchart of literature research (Moher et al. 2009; Page et al. 2021).

A flowchart of the literature research and review process is reported in Fig. 1. To evaluate the quality of the papers included, an assessment grid was used. The grid included nine major criteria with an assigned sub-score value depending on the quality of the assessment and reporting in original studies. We gave all items identified for a given criterion equal weight and the final quality was ranked high (11 to 16), moderate (5 to 10), and low (0 to 4) based on the total score, calculated as sum of the nine sub-scores (Table S3). Two reviewers (A.S. and F.B.) rated each study independently. The results were then compared, and all discrepancies were discussed and solved by consensus. In addition to standard study descriptors and method parameters, we extracted limitations/challenges in 2 ways: (i) limitations explicitly reported by the study authors and (ii) constraints inferred from the reported monitoring workflow (eg absence of speciation, fraction mismatch, potential redox artifacts, incomplete contextual determinants). Two reviewers independently coded these items into predefined thematic domains; discrepancies were resolved by consensus. The coded items are summarized in Table S8.

## Results and discussion

### General description of the reviewed studies

In total, this review included 79 publications (Fig. 1; Table S2). According to the evaluation criterion adopted and described in the section, the majority (*n* = 59, equal to 74.7% of the total) of the studies were of high quality, while the others were of moderate quality (*n* = 20, equal to 25.3% of the total). Details of the indices used for the calculation of quality are given in the Table S3 as well as the result of the judgement given based on the calculated indices (Table S4). Summary tables of the reviewed articles, organized by year of publication (Table S5) and geographical distribution (Table S6) are reported in the Supplementary materials. Twenty-nine of the 79 included studies (36.7%) analytically determined airborne Cr(VI), either alone or together with total chromium, as detailed in Table S2. More in detail, *n* = 4 measuring Cr(VI) only (Cena et al. 2015; Pesch et al. 2015; Bennett et al. 2016; Wang et al. 2017) and *n* = 25 reporting Cr(VI) alongside total Cr/total metals (Cate et al. 2014; Cena et al. 2014; Martin et al. 2015; Ščančar et al. 2015; Vincent et al. 2015; Järvelä et al. 2016; Laitinen et al. 2017; Nafti et al. 2017; Stanislawska et al. 2017; Berlinger and Harper 2018; Pesch et al. 2018; Keyter et al. 2019; Zendehdel et al. 2019; Bau et al. 2020; Shaw et al. 2020; Pourbakhshi et al. 2021; Oginawati et al. 2021; Lehnert et al. 2022; Spinazzè et al. 2022; Viegas et al. 2022; Gu et al. 2023; Jafari et al. 2023; Soltanpour et al. 2023; van Ree et al. 2023; Jiang et al. 2024), whereas 48 studies measured chromium as total Cr/total metals without speciation (Chen et al. 2014; de Oliveira Galvão et al. 2014; Julander et al. 2014; Lau et al. 2014; Lehnert et al. 2014; Wu and Liu 2014; Decharat 2015; Hamzah et al. 2016; Huang et al. 2016; Kettelarij et al. 2016; Miettinen et al. 2016; Nurul et al. 2016; Graff et al. 2017; Ogundele et al. 2017; Park et al. 2017; Sikder et al. 2017; Sakebayeva et al. 2018; Yang et al. 2018a, 2018b; Arsal Yıldırım et al. 2020; Kalteh et al. 2020; Kamaludin et al. 2020; Mehrifar et al. 2020; Onat et al. 2020; Dueck et al. 2021; Gerding et al. 2021; Newton et al. 2021a, 2021b; Vattanasit et al. 2021; Wu et al. 2021; Galarneau 2022; Kato et al. 2022; Mitra and Das 2022; Oddone et al. 2022; Eriksen Hammer et al. 2022; Hanser et al. 2022; Sepahi Zoeram et al. 2022; Wang et al. 2022; Gravel et al. 2023; Hamid et al. 2023; Khalili and Nasrabadi 2023; Khoshakhlagh et al. 2023; Lee et al. 2023; López et al. 2023; Newton et al. 2023; Pourhassan et al. 2024; Sarwar et al. 2023; Ghobakhloo et al. 2024)); in 2 studies, the Cr species was unclear or derived as a secondary estimate (Peters et al. 2015; Engelsman et al. 2019). Across field studies, speciated Cr(VI) was commonly quantified in the inhalable fraction (or as total suspended particles when inhalable conventions were not explicitly reported), although some sector-specific investigations also used respirable sampling (eg stainless-steel welding studies). Table 1 summarizes sampling strategies and particulate fractions across the reviewed evidence base.

**Table 1:** Overview of sampling strategies and particulate size fractions adopted in the reviewed field studies, grouped by sampling approach and fraction (EN 481 conventions).

Sampling technique	Particulate fraction	References
Stationary/fixed site sampling	TSP/Inhalable	Laitinen et al. (2017); Shaw et al. (2020); Hamid et al. (2023)
	Respirable	Vattanasit et al. (2021)
	TSP + Respirable	Lee et al. (2023)
	PM_10_	n.a.
	PM_2.5_	Ogundele et al. (2017); Sikder et al. (2017); Arsal Yıldırım et al. (2020); Mitra and Das (2022)
	Size-fractionated PM (different fraction)	n.a.
	Size fractionated PM (including nanometric particles)	Keyter et al. (2019); López et al. (2023)
	n.a.	Cate et al. (2014); Engelsman et al. (2019)
Personal sampling	TSP/Inhalable	Wu and Liu (2014); Kettelarij et al. (2016); Mehrifar et al. (2020); Newton et al. (2021a, 2021b); Oginawati et al. (2021); Viegas et al. (2022); Khalili and Nasrabadi (2023); Soltanpour et al. (2023); van Ree et al. (2023); Jiang et al. (2024)
	Respirable	Lehnert et al. (2014); Hamzah et al. (2016); Nurul et al. (2016); Pesch et al. (2018); Kamaludin et al. (2020)
	Inhalable + Respirable	Yang et al. (2018a, 2018b); Onat et al. (2020); Viegas et al. (2022)
	PM_2.5_	de Oliveira Galvão et al. (2014), Wu et al. (2021)
	Size-fractionated PM (different fraction)	n.a.
	n.a.	Chen et al. (2014); Lau et al. (2014); Zendehdel et al. (2019); Kalteh et al. (2020); Sepahi Zoeram et al. (2022); Gravel et al. (2023); Jafari et al. (2023); Khoshakhlagh et al. (2023); Pourhassan et al. (2024)
Stationary/fixed site Sampling and personal sampling	TSP/Inhalable	Julander et al. (2014); Cena et al. (2015); Vincent et al. (2015); Järvelä et al. (2016); Stanislawska et al. (2017); Dueck et al. (2021); Hanser et al. (2022); Wang et al. (2022)
	Respirable	Eriksen Hammer et al. (2022)
	Inhalable + Respirable	Stanislawska et al. (2017); Gerding et al. (2021); Lehnert et al. (2022); Oddone et al. (2022)
	PM10	Newton et al. (2021a, 2021b)
	PM_2.5_	Ghobakhloo et al. (2024)
	Size fractionated PM (including nanometric particles)	Miettinen et al. (2016); Graff et al. (2017)
	n.a.	Decharat (2015); Bennett et al. (2016)

References listed in each cell indicate studies that collected samples using the corresponding strategy/fraction; some studies appear in multiple cells when multiple fractions were measured. TSP = Total suspended particles; n.a.: information not available and/or details not further described in the reviewed articles. Note: In several large databases/campaigns (eg MEGA-derived analyses), Cr(VI) is quantified primarily in the inhalable fraction. Where studies report Cr(VI) and total Cr in different fractions and/or use both personal and stationary sampling, they are listed in multiple cells to reflect the sampling design.

### Field studies

Most of the articles reviewed in this study (*n* = 61) are field studies, in which researchers collected data directly from real-world occupational environments. These studies offer valuable insights into actual exposure levels and the associated health risks linked to various industrial processes. The occupational sectors covered in the reviewed literature are notably diverse and are summarized in Table S7.

Welding of stainless steel consistently emerged as a key exposure scenario (Berlinger and Harper 2018). The WELDOX and WELDOX II studies demonstrated that airborne Cr(VI) concentrations varied with welding process, type of steel, and local ventilation, with stainless steel producing the highest levels (Lehnert et al. 2014; Pesch et al. 2018). Particle-size investigations in stainless-steel welding fumes showed that a substantial fraction of Cr is associated with the submicron/ultrafine fraction (Cena et al. 2015) supporting the relevance of size-selective and task-based assessment (Miettinen et al. 2016; Stanislawska et al. 2017). In WELDOX II, measured Cr(VI) concentrations in personal samples were in the order of 0.5 to 1.3 µg/m^3^ under typical conditions (Pesch et al. 2018). A comprehensive exposure assessment in a stainless-steel fabrication facility reported median personal airborne concentrations for total Cr, nickel (Ni), and manganese of 66 µg/m^3^ (range: 13 to 300 µg/m^3^), 29 µg/m^3^ (5.7 to 132 µg/m^3^), and 22 µg/m^3^ (1.5 to 119 µg/m^3^), respectively; notably, only ∼1% of total Cr was water-soluble and thus bioavailable (Newton et al. 2021a). Other sectors also presented measurable exposure: several exposure scenarios are discussed in a study by Pesch and colleagues (Pesch et al. 2015) that enabled the analysis of 3,659 real personal inhalable Cr(VI) measurements from the MEGA database (1994 to 2009). In the printing industry, airborne chromium was reported as total Cr (mean 7.2 µg/m^3^; range 1 to 12 µg/m^3^) (Decharat 2015). These values are not directly comparable with Cr(VI)-specific OELs because Cr(VI) was not analytically determined; therefore, any regulatory comparison should be interpreted with caution. Risk factors included inadequate personal protective equipment (PPE) use and poor hygienic practices (Decharat 2015). In French battery recycling plants, although cadmium (Cd), cobalt (Co), and Ni dominated exposure profiles, Cr was also detected in inhalable dust, with maximum concentrations up to 19.3 µg/m^3^ in maintenance tasks (Hanser et al. 2022). Altogether, these findings confirm that relevant Cr(VI) exposures occur in welding, electroplating, surface treatment, ferrochromium production, stainless-steel fabrication, printing, and recycling. Exposure levels are heterogeneous, reflecting process-specific determinants and highlighting the importance of targeted preventive measures across sectors (Cena et al. 2015; Decharat 2015; Sakebayeva et al. 2018; Mehrifar et al. 2020; Newton et al. 2021a; Hanser et al. 2022).

The reviewed studies consistently demonstrated that occupational exposure to Cr(VI) varied significantly across industrial sectors and specific job tasks. One of the most comprehensive investigations—the HBM4EU Chromates study—evaluated Cr(VI) exposure among workers in nine European countries. Air monitoring revealed the highest concentrations of Cr(VI) in activities such as painting, thermal spraying, and especially bath plating, while welding and machining generated lower, yet still notable, levels. A strong correlation between inhalable and respirable fractions confirmed the presence of consistent airborne contamination. Additionally, dermal wipe samples indicated substantial hand exposure, particularly in plating and welding operations, highlighting the relevance of combined exposure routes (Santonen et al. 2022; Viegas et al. 2022). In addition to welding, electroplating and surface-treatment operations were consistently identified as high-risk activities for Cr(VI) exposure (Viegas et al. 2022; Jiang et al. 2024). In ferrochromium and stainless-steel production, airborne monitoring documented exposure to dust containing total Cr and, where speciated, Cr(VI), particularly during smelting and raw material handling (Järvelä et al. 2016). Other sectors also presented measurable chromium exposure: in the printing industry, airborne chromium was reported as total Cr (mean 7.2 µg/m^3^; range 1 to 12 µg/m^3^) and should not be compared directly with Cr(VI)-specific OELs (Decharat 2015); in French battery-recycling plants, Cr was detected in inhalable dust, with maxima up to 19.3 µg/m^3^ in maintenance tasks (Hanser et al. 2022). Overall, the field studies reviewed employed a range of methodologies to assess exposure, including environmental monitoring, biological sampling, and questionnaires.

In terms of environmental monitoring, studies evaluated airborne chromium in particulate matter through fixed-site, personal, or combined sampling approaches and across different particle-size fractions. Importantly, methods differed depending on whether Cr(VI) was analytically determined as such or whether chromium was measured as total Cr/total metals. Cr(VI)-specific determinations most commonly followed ISO 16740 or the NIOSH 7600-series/OSHA ID-215 approaches (alkaline extraction and 1,5-diphenylcarbazide colorimetry), whereas total metals were typically quantified after acid digestion using ICP-based or atomic-absorption techniques (eg NIOSH 7300-series or IFA 7808, without Cr speciation). Gravimetric methods and size-selective sampling conventions (eg EN 481 fractions) were frequently used to support the interpretation of exposure determinants. A concise mapping of the sampling and analytical approaches is provided in Table 2, while study-level method details extracted under the quality criteria are summarized in Table S8.

**Table 2:** Overview of target endpoints and corresponding sampling/analytical approaches used in the reviewed studies, distinguishing Cr(VI)-specific methods from total chromium/total metals and supporting particle metrics.

Target/endpoint	Typical sampling matrix/fraction	Common methods/standards (examples)	Key notes/limitations
Cr(VI) in airborne PM (speciated)	Airborne particles on filters (typically inhalable or total; PVC/MCE)	ISO 16740; NIOSH 7600/7604/7605; OSHA ID-215; IFA 6555	Alkaline extraction + DPC colorimetry or IC-based methods; risk of Cr(III)<−>Cr(VI) interconversion; requires preservation and artefact control.
Total Cr/total metals in airborne PM	Airborne particles on filters (inhalable/respirable/PM_2.5_/PM10; various samplers)	NIOSH 7300/7303/7304; NIOSH 7024; ISO 15202; IFA 7808; EPA 6010B	Quantifies elemental chromium without speciation; results are not directly comparable with Cr(VI) OELs; useful for context and determinants.
PM mass/size metrics (supporting exposure assessment)	Gravimetric PM (TSP/PM10/PM_2.5_; inhalable/respirable conventions) and/or real-time particle metrics	EN 481 size-selective fractions; NIOSH 0500/0600; OPC/CPC/SMPS/ELPI	Supports interpretation of size fractions and task peaks; not specific to Cr(VI) unless coupled with chemical analysis.
Biomonitoring (internal dose)	Urine (most common), occasionally blood/serum	Urinary Cr by ICP-MS/ICP-OES/AAS	Reflects integrated chromium uptake (not Cr(VI)-specific); susceptible to background sources and co-exposures; best interpreted alongside airborne Cr(VI) monitoring.

It is important to acknowledge several limitations in field-based exposure assessments. A major challenge lies in distinguishing between Cr species, as most studies report total Cr rather than Cr(VI), thereby limiting the accuracy of risk evaluations. In this review (79 included studies), 29 studies quantified Cr(VI) in air (either alone or alongside total chromium/metals), whereas 48 reported only total chromium/metals; the remaining publications were model-/database-based or lacked sufficient detail for unambiguous speciation classification (Table S2). The inherent instability of Cr(VI) further complicates measurement reliability, with uncertainties arising from sampling devices, filter types, sampling duration, and storage conditions (Spinazzè et al. 2022). Particle size is another critical factor, as ultrafine particles (<100 nm) may pose greater health risks but are frequently underestimated due to instrumental limitations (Cena et al. 2014; Miettinen et al. 2016; Stanislawska et al. 2017). To address these gaps, several studies have integrated environmental and biological monitoring approaches, analyzing Cr levels in blood, urine, or both matrices (Hanser et al. 2022; Viegas et al. 2022). These investigations revealed that correlations with biological markers are influenced by inter-individual variability and the use of PPE. Additional studies have included dermal exposure assessments, recognizing the relevance of multiple exposure pathways (Viegas et al. 2022). Complementary strategies, such as activity-based exposure models and statistical analyses, have also been employed to identify key exposure determinants and inform targeted preventive measures (Pesch et al. 2018).

### Laboratory studies, simulations, and new methodological approaches

Part of the articles reviewed in this paper (*n* = 13) concerned laboratory simulations, or the development and refinement of new analytical methods (Cena et al. 2014; Ščančar et al. 2015; Huang et al. 2016; Mariem Nafti Radhouane Chakroun and Nouaïgui 2017; Park et al. 2017; Wang et al. 2017; Berlinger and Harper 2018; Bau et al. 2020; Pourbakhshi et al. 2021; Kato et al. 2022; Spinazzè et al. 2022; Gu et al. 2023; Newton et al. 2023). These studies described different analytical methodologies for the determination of Cr(VI) and other metals in environmental matrices, with a focus on welding fume emissions. Different methods have been compared, including liquid chromatography-inductively coupled plasma mass spectrometry (HPLC-ICP-MS), atomic absorption spectrometry, and portable X-ray fluorescence spectrometry, evaluating limits of detectability, accuracy, and precision. Different studies have investigated variable aspects of particle exposure in industrial settings. Some focused on quantifying ultrafine metal particles in welding fumes, while others examined the extraction of Cr(VI) from matrices rich in trivalent chromium Cr(III). One study specifically analyzed particle emissions from an additive manufacturing process, emphasizing their chemical composition and size distribution (Cena et al. 2014). Among the research on welding fumes, a notable study explored the size and composition of emitted particles to assess the potential health risks associated with exposure. The findings highlighted a critical limitation in current exposure assessment methods, which often fail to differentiate between ultrafine and larger particles. This is particularly concerning, as insoluble ultrafine particles may exhibit distinct toxicological behaviors due to their increased surface area and reactivity (Cena et al. 2014). The same study proposed a novel approach using a respiratory nanoparticle deposition (NRD) sampler, designed to selectively collect ultrafine particles in a manner that simulates their deposition within the human respiratory tract. The findings revealed that a substantial fraction of metals—including Cr, Mn, and Ni—were present in particles smaller than 300 nm. These results underscore the critical importance of accounting for ultrafine particles in exposure assessments, given their distinct deposition behavior and potential for enhanced toxicological effects (Cena et al. 2014).

The speciation of metals in welding fumes, in particular Cr(VI), was also an area of interest (Cena et al. 2014; Ščančar et al. 2015; Spinazzè et al. 2022). Analytical protocols have been developed to accurately determine Cr(VI) in the presence of high concentrations of Cr(III) (Spinazzè et al. 2022), which is more common, but less toxic. A liquid chromatography–inductively coupled plasma mass spectrometry (LC-ICP-MS) analytical method, combined with an isotope-enriched spike addition technique, was proposed to investigate Cr(III)/Cr(VI) interconversions during the extraction process. These protocols typically involve an alkaline extraction step, followed by analytical techniques such as ion chromatography (IC) coupled with ICP-MS, enabling accurate speciation and quantification of Cr forms (Cena et al. 2014; Ščančar et al. 2015; Spinazzè et al. 2022). To characterize particle emissions from additive manufacturing processes, one study (Bau et al. 2020) used a combination of real-time instruments and samplers positioned at various locations, including the source, near-field, far-field, and operator to determine the mass size distribution of aerosols and to measure particle number and size concentrations. The results indicated that a significant part of the mass concentration was related to the ultrafine fraction and that more than 95% could be linked to the respirable fraction.

In addition to particle size characterization, studies have also examined the elemental composition of welding fumes using various techniques, such as induction plasma atomic emission spectrometry (ICP-OES) (Ščančar et al. 2015), electrothermal atomic absorption spectrometry (Mariem Nafti Radhouane Chakroun and Nouaïgui 2017), scanning electron microscopy coupled with energy dispersive X-ray spectroscopy (SEM-EDS) (Kato et al. 2022), and portable X-ray fluorescence spectrometry (FP-XRF) (Park et al. 2017; Newton et al. 2023). These techniques showed satisfactory performance in providing valuable information on the elemental composition of welding fumes, which can be used to assess potential health risks. A study (Newton et al. 2023) compared the performance of FP-XRF with ICP-MS for the determination of metals in welding fumes: results of the study showed that FP-XRF could provide accurate estimates of personal metal exposures but overestimated the mass of some metals. However, the linearity of the response suggested that appropriate correction factors could be developed. Finally, an interlaboratory comparison (Berlinger and Harper 2018) evaluated a method to determine the water-soluble component of welding fume samples. The results showed good repeatability within the laboratory, but inter-laboratory reproducibility was less satisfactory, although within acceptable limits. This study highlights the importance of standardized methods for assessing the bio-available fraction of metals in welding fumes.

Among innovative techniques for characterizing occupational exposure to Cr(VI), a few studies (*n* = 5) employed modeling approaches (Pesch et al. 2015; Peters et al. 2015; Galarneau 2022; Wang et al. 2022; Pourhassan et al. 2024). Wang and colleagues (Wang et al. 2022) applied a near-field/far-field model with Bayesian decision analysis to estimate welders' exposure, showing probabilities above action levels. An exposure matrix for welders was developed and validated against experimental data (Galarneau 2022). CAREX, a large-scale model for carcinogen exposure in Canada, was adapted using EU expert inputs (Peters et al. 2015). A further study applied multiple risk assessment methods to welding fumes (Pourhassan et al. 2024). All reviewed modeling studies pointed to significant health risks for exposed workers and demonstrated the potential of mathematical models, Bayesian analysis, and exposure matrices as alternatives to direct measurement. Their main advantage is the ease of application, but limitations include incomplete long-term data, variability of work scenarios, and the need for further validation (Table S2). Ultimately, exposure assessment for carcinogens must rely on scientifically robust data with clearly defined uncertainty (Kromhout 2016).

Although the studies just discussed focus on laboratory simulations and the development of new technologies, some were also accompanied by measurements in real workplace scenarios. For instance, while the NRD sampler was first tested in controlled conditions, other investigations assessed particle concentrations directly at operator workstations in additive manufacturing centers (Bau et al. 2020), or combined air and urine analyses in electroplating workers (Pourbakhshi et al. 2021). Additional field applications include air monitoring in a chromite production facility using an electrochemical sensor (Gu et al. 2023), and characterization of dust from high-velocity oxygen fuel spraying in a Chinese company (Huang et al. 2016). Likewise, a newly developed analytical method for Cr(VI) was validated through personal sampling in a leather tannery (Spinazzè et al. 2022).

### Lessons learnt, critical issues, and perspectives

This review highlights several critical issues in assessing occupational exposure to Cr(VI).

Field studies relied on diverse methods, primarily air monitoring complemented by contextual determinants; a subset of studies also included surface and biological sampling showing the need for an integrated approach that considers multiple exposure routes (Pesch et al. 2018; Shaw et al. 2020; Newton et al. 2021b; Oddone et al. 2022; Viegas et al. 2022; Ghobakhloo et al. 2024; Jiang et al. 2024).

However, significant analytical challenges remain. Measuring Cr(VI) is complicated by its instability and by interconversion with Cr(III) during standard hot alkaline extractions, which may lead to over- or underestimation (Spinazzè et al. 2022). This flaw is particularly critical for samples in which the amount of Cr(III) can be predominant on that of Cr(VI). The lack of reliable speciation methods undermines accurate risk assessment. Data availability is also uneven: inhalable fractions of Cr(VI) are less frequently characterized than total Cr (Pesch et al. 2018; Shaw et al. 2020), and conventional particle-size–specific analyses are costly and time-consuming (Park et al. 2017), thus rapid and inexpensive analytical techniques are urgently needed to allow more frequent monitoring and timely interventions (Cate et al. 2014; Jafari et al. 2023; López et al. 2023; van Ree et al. 2023). Variability across tasks, materials, and collective and PPE effectiveness further complicates exposure assessment, making contextual information essential (Viegas et al. 2022).

Moreover, risk management cannot rely on PPE alone: training, maintenance of local exhaust ventilation, and workplace organization (eg storage of contaminated clothing or equipment; activity organization; work procedures) are crucial determinants (Lehnert et al. 2022; Khalili and Nasrabadi 2023; Khoshakhlagh et al. 2023). Interlaboratory variability remains high, stressing the need for harmonized and validated analytical methods (Berlinger and Harper 2018). Previous monitoring campaigns have shown potential in reducing exposure, though their effectiveness depends on transparent communication and follow-up actions (Gatto et al. 2010; Hessel et al. 2021).

Where biomonitoring data were available in included studies, they may support interpretation of cumulative uptake; however, interpretation requires robust correlations, careful consideration of toxicokinetic across exposure routes, and attention to background sources.

The recent HBM4EU Chromates Study findings show a strong association between airborne Cr(VI)/Cr metrics and urinary total chromium, but with sector-dependent slopes (eg welders presenting lower urinary chromium levels than platers at comparable inhalation exposure), consistent with species-dependent kinetics and the influence of non-inhalation determinants; therefore, urinary chromium should be interpreted as a nonspecific marker and contextualized with concurrent air monitoring and task/control information (Santonen et al. 2022; Viegas et al. 2022). Alternative biomarkers (eg blood/RBC chromium and EBC chromium) have been explored in the same framework and may complement interpretation in selected contexts but require careful validation and standardized reporting (Ndaw et al. 2022; Santonen et al. 2022, 2023).

Speciation stability and artifact control remain a central challenge. In Cr(III)-rich particulate matrices (eg welding fumes), Cr(VI) may be reduced during sampling, transport or extraction, whereas Cr(III) can be oxidized under strongly alkaline conditions; therefore, protocols need explicit preservation steps and documented recovery/artifact tests. Where possible, methods aligned with ISO 16740 or equivalent validated approaches (eg NIOSH 7600/7604/7605, OSHA ID-215, IFA 6555) should be prioritized, and results should clearly indicate the sampled fraction (inhalable vs respirable) and reporting unit (as Cr(VI) vs total Cr).

Biomonitoring (urinary Cr) can complement air monitoring but should be interpreted cautiously: urinary Cr reflects integrated Cr uptake from all routes and is not specific to Cr(VI). Background dietary/environmental sources and co-exposures can dominate at low occupational exposures. Consequently, urinary Cr should not be interpreted as a surrogate of airborne Cr(VI) unless contextual exposure and speciation data are available and is most informative when paired with concurrent airborne Cr(VI) measurements and detailed task/controls information. Integration of biomonitoring data with environmental data can capture cumulative exposure, but interpretation requires robust correlations and careful consideration of toxicokinetic across exposure routes (Gatto et al. 2010; Hessel et al. 2021). Where biomonitoring data were available, they contributed to characterizing internal Cr uptake; however, their interpretability—particularly in welding settings—remains limited by non-specificity for Cr(VI), background sources, timing of sampling, and species-dependent toxicokinetics. Several studies have shown weak or inconsistent correlations between urinary Cr and airborne Cr(VI) among welders, reflecting the influence of fume solubility, mixed exposure routes, and dermal contamination (Stanislawska et al. 2020; Santonen et al. 2022; Viegas et al. 2022). The HBM4EU Chromates Study similarly reported lower urinary Cr levels in welders than in platers at comparable inhalation exposure, underscoring the need to contextualize biomonitoring data with concurrent air measurements and task information (Santonen et al. 2022; Viegas et al. 2022).

Consistent with this evidence, Birk et al. (2006) observed only modest air–urine correlations in stainless-steel welders and substantial inter-individual variability linked to welding technique and skin contamination. Their study also identified a dose–response trend between urinary Cr and early adverse effect—such as nasal irritation and oxidative stress markers—although associations weakened after adjustment for smoking and co-exposures, highlighting the limited specificity of urinary Cr as an indicator of Cr(VI)-related health risk. Alternative biomarkers (eg blood/RBC chromium, exhaled breath condensate chromium) may complement urinary chromium in selected contexts, but require further validation, harmonized analytical methods, and standardized reporting (Ndaw et al. 2022; Santonen et al. 2023). Overall, biomonitoring can support exposure assessment, but its standalone use in welders is constrained by toxicokinetic complexity and confounding factors, reinforcing the need for integrated interpretation.

Temporal trends should also be interpreted cautiously: although several industrialized settings report declining Cr(VI) levels over time, improvements may partly reflect changes in monitoring strategies, analytical workflows, or process substitution rather than true reductions in exposure. Multi-country initiatives based on harmonized protocols offer a more robust basis for international benchmarking. In this regard, the HBM4EU Chromates Study provides a coordinated framework across several European countries, using standardized approaches for exposure characterization and biomonitoring, thereby supporting more reliable between-setting comparisons (Santonen et al. 2022, 2023; Viegas et al. 2022).

We did not perform a formal cross-country comparison of Cr(VI) exposure levels in this review, because differences in sampling conventions (inhalable/respirable/total), speciation capability and stability controls, task structures and control technologies, and regulatory contexts can substantially limit comparability and may lead to methodological artifacts. Nevertheless, multi-country initiatives based on harmonized protocols provide a more suitable framework for international benchmarking; for example, the HBM4EU Chromates Study offers a broader perspective across several European countries using a common approach to exposure characterization and biomonitoring, supporting more robust between-setting comparisons (Santonen et al. 2022, 2023; Viegas et al. 2022). Cross-country comparisons of occupational Cr(VI) exposure are constrained by differences in sampling conventions (inhalable/respirable/total), speciation capability, task structure and control technologies, as well as regulatory contexts. Similarly, time trends within single programs or campaigns should be interpreted cautiously because monitoring strategies and analytical workflows may change over time.

Looking ahead, strengthening analytical standardization, systematically collecting contextual determinants, and combining environmental, biological, and modeling approaches will be crucial to improve exposure and risk assessment. Future studies should also (i) accurately distinguish between different chemical species of metals; (ii) employ tools capable of measuring particle size distribution, especially ultrafine particles; and (iii) gather contextual data on collective and PPE use and work activities to refine exposure characterization and preventive strategies.

## Conclusions

The study analyzed, through a systematic review of the literature, the available procedures for assessing occupational exposure to Cr(VI) through environmental monitoring. Some included studies reported biomonitoring data alongside air monitoring; however, the primary contribution of this review concerns environmental monitoring and Cr(VI) speciation challenges. Overall, the review supports integrated exposure assessment frameworks that combine fit-for-purpose airborne Cr(VI) monitoring with contextual determinants (tasks, materials, controls); where available, complementary surface and biological measurements may support interpretation, but were not systematically reviewed as primary endpoints. The analysis of 79 studies, conducted in different work settings, allowed to identify some key issues. In a nutshell, the most pressing issues are the difficulty of distinguishing between Cr species, the instability of Cr(VI) during sampling and analysis, and the limited availability of reliable speciation methods. These factors compromise the accuracy of exposure assessment and hinder the estimation of health risks. In addition, data on particle size distribution remains scarce, particularly for ultrafine particles, despite their toxicological relevance. Innovative methods—such as nanoparticle focused monitoring tools, improved analytical protocols for Cr(VI) in complex matrices, and modeling approaches—show promise, but require further validation. Equally important is the collection of contextual information on determinants such as work practices as well as collective and PPE use, which strongly influences actual exposure levels. Overall, the review underscores the importance of advancing analytical accuracy, harmonizing methods, and strengthening integrated exposure assessment frameworks. Future research should (i) accurately distinguish between different chemical species of metals; (ii) adopt sampling and analytical techniques capable of characterizing particle size distribution, with particular attention to ultrafine fractions; and (iii) systematically collect contextual data on work activities and preventive measures adopted. By addressing these gaps, future studies will improve risk assessment and support more effective prevention strategies to protect Cr(VI) exposed workers.

## Supplementary Material

wxag047_Supplementary_Data

## Data Availability

All data generated or analyzed during this study are included in this published article and its Supplementary information files.
