# Supplementary material for: Occupational exposure to hexavalent chromium: a systematic review of environmental monitoring methods and analytical advances
Source: Ann Work Expo Health. 2026 Jun 22;70(5):wxag047. doi: 10.1093/annweh/wxag047 (PMC13285725; doi:10.1093/annweh/wxag047)
Supplement: wxag047_Supplementary_Data [file wxag047_supplementary_data.pdf]

Occupational Exposure to Hexavalent Chromium: A Systematic Review of Environmental Monitoring Methods and Analytical Advances

Andrea Spinazzè[1,*], Francesca Borghi[1,2], Carolina Zellino[1], Veronica Prina[1], Andrea Cattaneo[1], Sandro Recchia[1], Carlo Dossi[3], Salvatore Della Notte[4], Veruscka Leso[4], Ivo Iavicoli[5,6,#], Domenico Maria Cavallo[1#].

[1] Department of Science and High Technology, University of Insubria, via Valleggio 11, 22100 Como (Italy)

2 Department of Medical and Surgical Sciences, University of Bologna, via P. Palagi 9, 40138 Bologna (Italy)

[3] Department of Theoretical and Applied Sciences, University of Insubria, Via O. Rossi, 9, 21100 Varese (Italy)

[4] Department of Public Health, Section of Occupational Medicine, University of Naples Federico II, Via S. Pansini 5, 80131 Naples (Italy)

[5] Dipartimento di Sicurezza e Bioetica, Catholic University of Sacred Heart, Largo Francesco Vito 1, 00168 Rome (Italy)

[6] Fondazione Policlinico Universitario A. Gemelli, IRCCS, Largo Agostino Gemelli 8, 00168 Rome (Italy)

Co-Senior Authors

*** Corresponding Author**

Andrea Spinazzè - Department of Science and High Technology, University of Insubria. Via Valleggio 11, 22100 Como (CO) - Italy; Telephone: +39 031 2386629; Email: andrea.spinazze@uninsubria.it

SUPPLEMENTARY MATERIALS

Table S1. Search query arranged for each database (last search: March 2024).

Database	Search query
**Scopus**	TITLE-ABS-KEY ("hexavalent chromium" OR chromium* OR "chromates" OR chromate* OR "Cr(VI)" OR "Cr(6+)" OR "chromium (VI)") AND TITLE-ABS-KEY ("occupational exposure" OR "exposure evaluation" OR "exposure assessment" OR sampl* OR monitor* OR "determination") AND TITLE-ABS-KEY (occupation* OR "work environment") AND PUBYEAR > 2013
**Web of Science**	("hexavalent chromium"[Title/Abstract] OR chromium*[Title/Abstract] OR "chromates"[Title/Abstract] OR chromate*[Title/Abstract] OR "Cr(VI)"[Title/Abstract] OR "Cr(6+)"[Title/Abstract] OR "chromium (VI)"[Title/Abstract]) AND ("occupational exposure"[Title/Abstract] OR "exposure evaluation"[Title/Abstract] OR "exposure assessment"[Title/Abstract] OR sampl*[Title/Abstract] OR monitor*[Title/Abstract] OR "determination"[Title/Abstract]) AND (occupation*[Title/Abstract] OR "work environment"[Title/Abstract]) AND ("2014/01/01"[PDAT] : "2025/12/31"[PDAT])
**PubMed**	TS=("hexavalent chromium" OR chromium* OR "chromates" OR chromate* OR "Cr(VI)" OR "Cr(6+)" OR "Cr(III)" OR "chromium (VI")) AND TS=("occupational exposure" OR "exposure evaluation" OR "exposure assessment" OR sampl* OR monitor* OR air* OR "determination") AND TS=(occupation* OR "work environment") AND PY=2014-2024

Table S2. Complete list of papers found suitable and reviewed in this study and their quality (see table S3 and S4 for details on quality score).

Title	Publication Year	Reference	Cr species	Quality Assess-ment	Quality Assessment score
A Novel Method for Assessing Respiratory Deposition of Welding Fume Nanoparticles	2014	(Cena *et al.*, 2014)	Cr(VI)+Total	Moderate	10
Chromium Exposure and Hygienic Behaviors in Printing Workers in Southern Thailand	2015	(Decharat, 2015)	Total	High	14
Health risk assessment of heavy metals in exposed workers of municipal waste recycling facility in Iran	2024	(Ghobakhloo *et al.*, 2024)	Total	High	12
Long-term metal fume exposure assessment of workers in a shipbuilding factory	2022	(Wang, Kuo, and Wang, 2022)	Total	High	14
Relationships between inhalable and total hexavalent chromium exposures in steel passivation, welding and electroplating operations of Ontario	2020	(Shaw *et al.*, 2020)	Cr(VI)+Total	High	13
On the Determination of Cr(VI) in Cr(III)-Rich Particulates: From the Failure of Official Methods to the Development of an Alternative Protocol	2022	(Spinazzè *et al.*, 2022)	Cr(VI)+Total	High	14
Simultaneous speciation analysis of chromate, molybdate, tungstate and vanadate in welding fume alkaline extracts by HPLC-ICP-MS	2015	(Ščančar *et al.*, 2015)	Cr(VI)+Total	Moderate	10
Determination of Chromium (VI) in Airborne Particulate Matter by Electrothermal Atomic Absorption Spectrometry	2017	(Mariem Nafti Radhouane Chakroun and Nouaïgui, 2017)	Cr(VI)+Total	High	11
Dispersive Liquid-Liquid Microextraction Followed by Solidified Floating Organic Drop for Hexavalent Chromium Determination: a Method for Occupational and Environmental Exposure Monitoring for Heavy Metals	2021	(Pourbakhshi *et al.*, 2021)	Cr(VI)+Total	Moderate	8
Characterizing particle emissions from a direct energy deposition additive manufacturing process and associated occupational exposure to airborne particles	2020	(Bau *et al.*, 2020)	Cr(VI)+Total	High	11
Laboratory comparison of field portable X-ray fluorescence spectrometer (FP-XRF) and inductively coupled plasma mass spectrometry (ICP-MS) for determination of airborne metals in stainless steel welding fume	2023	(Newton *et al.*, 2023)	Cr(VI)+Total	Moderate	10
Interlaboratory comparison for the determination of the soluble fraction of metals in welding fume samples	2018	(Berlinger and Harper, 2018)	Cr(VI)+Total	Moderate	9
Construction and Calibration of an Exposure Matrix for the Welding Trades	2022	(Galarneau, 2022)	Total	Moderate	6
Analytical method using SEM-EDS for metal elements present in particulate matter generated from stainless steel flux-cored arc welding process	2022	(Kato *et al.*, 2022)	Total	Moderate	9
Appraising the characteristics of particulate matter from leather tanning micro-environments, their respirational risks, and dysfunctions amid exposed working cohorts	2023	(Sarwar *et al.*, 2023)	Total	Moderate	5
Assessment of occupational health risk due to inhalation of chemical compounds in an aircraft maintenance, repair, and overhaul company	2023	(Khalili and Nasrabadi, 2023)	Total	High	13
Assessment of the health risk related to exposure to ultrafine, fine, and total particulates and metals in a metal finishing plant	2020	(Onat *et al.*, 2020)	Total	High	12
Cancer risk assessment for occupational exposure to chromium and nickel in welding fumes from pipeline construction, pressure container manufacturing, and shipyard building in Taiwan	2018	(Yang *et al.*, 2018a)	Total	High	13

Carcinogenic and non-carcinogenic risk of exposure to metal fume in different types of welding processes	2023	(Soltanpour *et al.*, 2023)	Cr(VI)+Total	High	14
Cashew nut roasting: Chemical characterization of particulate matter and genotoxicity analysis	2014	(Galvão *et al.*, 2014)	Total	Moderate	9
Characterization of occupational smoke exposure among wildland firefighters in the midwestern United States	2021	(Wu *et al.*, 2021)	Total	High	11
Characterization of particle exposure in ferrochromium and stainless steel production	2016	(Järvelä *et al.*, 2016)	Cr(VI)+Total	High	13
Chemical pollutants in the respiratory zone of welders: Determination of concentrations and hazard analysis	2020	(Mehrifar, Zeverdegani, and Rismanchian, 2020)	Total	High	11
Coarse, fine and ultrafine particles arising during welding - Analysis of occupational exposure	2017	(Stanislawska *et al.*, 2017)	Cr(VI)+Total	High	13
Determinants of metals exposure to metalworking fluid among metalworkers in taiwan	2014	(Wu and Liu, 2014)	Total	High	12
Ultrasensitive Electrochemical Detection of Cr(VI) in the Air of Workplace Using the Bismuth Film Modified Electrode	2023	(Gu *et al.*, 2023)	Cr(VI)+Total	Moderate	10
HBM4EU Chromates Study: Determinants of Exposure to Hexavalent Chromium in Plating, Welding and Other Occupational Settings	2022	(Viegas *et al.*, 2022)	Cr(VI)+Total	High	13
Evaluating Measuring Techniques for Occupational Exposure during Additive Manufacturing of Metals: A Pilot Study	2017	(Graff *et al.*, 2017)	Total	Moderate	10
Exposure to hexavalent chromium in welders: Results of the WELDOX II field study	2018	(Pesch *et al.*, 2018)	Cr(VI)+Total	High	14
Exposure to metals and semivolatile organic compounds in Australian fire stations	2019	(Engelsman *et al.*, 2019)	Unclear	High	12
Formal recycling of e-waste leads to increased exposure to toxic metals: AN occupational exposure study from Sweden	2014	(Julander *et al.*, 2014)	Total	High	12
Health risk assessment of exposure to various vapors and fumes in a factory of automobile manufacturing	2023	(Khoshakhlagh *et al.*, 2023)	Total	High	13
Health risk assessment of inhalational exposure to heavy metals in drivers working in an urban desert city in the Middle East	2022	(Sepahi Zoeram *et al.*, 2022)	Total	High	14
Heavy metals in industrially emitted particulate matter in Ile-Ife, Nigeria	2017	(Ogundele *et al.*, 2017)	Total	Moderate	9
Hexavalent chromium still a concern in Sweden - Evidence from a cross-sectional study within the SafeChrom project	2023	(Jiang *et al.*, 2024)	Cr(VI)+Total	High	15
How to Reduce the Exposure of Welders to an Acceptable Level: Results of the InterWeld Study	2022	(Lehnert *et al.*, 2022)	Cr(VI)+Total	High	11
Human health risk assessment based on trace metals in suspended air particulates, surface dust, and floor dust from e-waste recycling workshops in Hong Kong, China	2014	(Lau *et al.*, 2014)	Total	High	13
Lung deposition versus inhalable sampling to estimate body burden of welding fume exposure: A pilot sampler study in stainless steel welders	2021	(Newton *et al.*, 2021b)	Total	High	12
Metal dust exposure and lung function deterioration among steel workers: an exposure-response relationship	2016	(Hamzah, Mohd Tamrin, and Ismail, 2016)	Total	High	14
Metal exposure of workers during recycling of electronic waste: a cross-sectional study in sheltered workshops in Germany	2021	(Gerding *et al.*, 2021)	Total	High	14
Neurological risk assessment of co-exposure to heavy metals (chromium and nickel) in chromium-electroplating workers	2019	(Zendehdel, Fazli, and Rezazadeh Azari, 2019)	Cr(VI)+Total	High	12
Occupational exposure to chrome VI compounds in french companies: Results of a national cam-	2015	(Vincent *et al.*, 2015)	Cr(VI)+Total	High	14

paign to measure exposure (2010-2013)					
Occupational exposure to nanoparticles at commercial photocopy centers	2015	(Martin *et al.*, 2015)	Cr(VI)+Total	High	12
Particle measurements of metal additive manufacturing to assess working occupational exposures: a comparative analysis of selective laser melting, laser metal deposition and hybrid laser metal deposition	2022	(Oddone *et al.*, 2022)	Total	High	11
Particle size and metal composition of gouging and lancing fumes	2019	(Keyter, Van Der Merwe, and Franken, 2019)	Cr(VI)+Total	High	14
Peak exposures to main components of ash and gaseous diesel exhausts in closed and open ash loading stations at biomass-fuelled power plants	2017	(Laitinen *et al.*, 2017)	Cr(VI)+Total	Moderate	9
Physicochemical Characteristics of Dust Particles in HVOF Spraying and Occupational Hazards: Case Study in a Chinese Company	2016	(Huang, Li, and Li, 2016)	Total	Moderate	8
Physicochemical Characterization of Aerosol Generated in the Gas Tungsten Arc Welding of Stainless Steel	2016	(Miettinen, Torvela, and Leskinen, 2016)	Total	Moderate	10
Reduction in welding fume and metal exposure of stainless steel welders: An example from the WELDOX study	2014	(Lehnert *et al.*, 2014)	Total	High	14
Reliability and validity of expert assessment based on airborne and urinary measures of nickel and chromium exposure in the electroplating industry	2014	(Chen *et al.*, 2014)	Total	Moderate	9
Risk analysis of inhaled hexavalent chromium (Cr6+) exposure on blacksmiths from industrial area	2021	(Oginawati *et al.*, 2021)	Cr(VI)+Total	High	13
Assessment and mapping of total suspended particulate and soil quality around brick kilns and occupational health issues among brick kilns workers in Pakistan	2023	(Hamid *et al.*, 2023)	Total	Moderate	8
Assessment of dust exposure in a steel plant in the eastern coast of peninsular Malaysia	2016	(Nurul, Shamsul, and Noor Hassim, 2016)	Total	High	13
Assessment of occupational exposure to fine particulate matter in dental prosthesis laboratories in Kocaeli, Turkey	2020	(Arsal Yıldırım, Pekey, and Pekey, 2020)	Total	High	12
Snapshot of cobalt, chromium and nickel exposure in dental technicians	2016	(Kettelarij *et al.*, 2016)	Total	High	14
Toxicity assessment of ash and dust from handmade gold jewelry manufacturing workshops in Bangladesh	2017	(Sikder *et al.*, 2017)	Total	Moderate	8
Welding Fume Exposure and Health Risk Assessment in a Cohort of Apprentice Welders	2021	(Dueck *et al.*, 2021)	Total	High	13
A field study on the respiratory deposition of the nano-sized fraction of mild and stainless steel welding fume metals	2015	(Cena *et al.*, 2015)	Cr(VI)	High	12
Characterisation of Particles Emitted during Laser Cutting of Various Metal Sheets and an Exposure Assessment for the Laser Operators	2022	(Eriksen Hammer *et al.*, 2022)	Total	High	13
Exposure to silica, arsenic, and chromium (Vi) in cement workers: A probability health risk assessment	2020	(Kamaludin *et al.*, 2020)	Total	High	13
Health risk assessment of metal fumes in an Iranian Mineral Salt company	2020	(Kalteh *et al.*, 2020)	Total	High	11
Hexavalent chromium and isocyanate exposures during military aircraft painting under crossflow ventilation	2016	(Bennett *et al.*, 2016)	Cr(VI)	High	11
Mass-size distribution and concentration of metals from personal exposure to arc welding fume in pipeline construction: A case report	2018	(Yang *et al.*, 2018b)	Total	High	13
Measurement of Airborne Particles and Volatile	2023	(Lee *et al.*,	Total	High	13

Organic Compounds Produced During the Heat Treatment Process in Manufacturing Welding Materials		2023)			
Occupational exposure to metals among battery recyclers in France: Biomonitoring and external dose measurements	2022	(Hanser *et al.*, 2022)	Total	High	13
Occupational health and safety risk assessment in chrome production	2018	(Sakebayeva *et al.*, 2018)	Total	Moderate	7
Occupational health and safety, metal exposures and multi-exposures health risk in Canadian electronic waste recycling facilities	2023	(Gravel *et al.*, 2023)	Total	High	14
Personal and area exposure assessment at a stainless steel fabrication facility: an evaluation of inhalable, time-resolved PM10, and bioavailable airborne metals	2021	(Newton *et al.*, 2021a)	Total	High	12
Rapid analysis of the size distribution of metal-containing aerosol	2017	(Park *et al.*, 2017)	Total	High	11
Rapid detection of transition metals in welding fumes using paper-based analytical devices	2014	(Cate *et al.*, 2014)	Cr(VI)+Total	High	12
Size-resolved chemical composition and toxicity of particles released from refit operations in shipyards	2023	(López *et al.*, 2023)	Total	High	11
Toluene and Heavy Metals in Small Automotive Refinishing Shops and Personal Protection of the Workers in Nakhon Si Thammarat, Thailand	2021	(Vattanasit *et al.*, 2021)	Total	High	14
Characterization of Particulate Fume and Oxides Emission from Stainless Steel Plasma Cutting	2017	(Wang *et al.*, 2017)	Cr(VI)	High	12
Health risk assessment of construction workers from trace metals in PM2.5 from Kolkata, India	2022	(Mitra and Das, 2022)	Total	High	12
Monte Carlo-based probabilistic risk assessment for cement workers exposed to heavy metals in cement dust	2023	(Jafari *et al.*, 2023)	Cr(VI)+Total	High	14
CAREX Canada: An enhanced model for assessing occupational carcinogen exposure	2015	(Peters *et al.*, 2015)	Secondary	Moderate	5
Airborne exposure to inhalable hexavalent chromium in welders and other occupations: Estimates from the German MEGA database	2015	(Pesch *et al.*, 2015)	Cr(VI)	High	11
Application of Multiple Occupational Health Risk Assessment Models for Metal Fumes in Welding Process	2023	(Pourhassan *et al.*, 2024)	Total	High	15
Emissions and Exposures Associated with the Use of an Inconel Powder during Directed Energy Deposition Additive Manufacturing	2023	(van Ree, du Preez, and du Plessis, 2023)	Cr(VI)+Total	High	12

Table S3. Quality assessment grid.

QUALITY CRITERIA		SCORE
Q1 - Were the aims of the study clearly defined?	Yes	2
	Enough	1
	No	0
Q2 - Study design	Field study	3
	Lab. Simulation	2
	Modeling	1
Q3 - Has the sample size been clearly defined?	Yes	1
	No	0
Q4 - Has the fraction of Cr investigated been clearly defined?	Yes	1
	No	0
Q5 - Have the sampling methods been clearly and comprehensively defined? *[Evaluate which of the following information states "Not available": (i) Sampling period (h; min); (ii) Instrumentation; (iii) Filter/substrate; (iv) Flow rate]*	0 information not available	4
	1 information not available	3
	2 information not available	2
	3 information not available	1
	4 information not available	0
Q6 - Was the sampling method followed reported	Yes	1
	No	0
Q7 - Have the analysis methods been clearly and completely defined? *[Evaluate which of the following information states "Not available":(i) Pre-analysis; (ii) Instrumentation / brief description of the method]*	0 information not available	2
	1 information not available	1
	2 information not available	0
Q8 - Has the analysis method followed been reported?	Yes	1
	No	0
Q9 - Are LOD (Limit of Detection) and/or LOQ (Limit of Quantification) reported?	Yes	1
	No	0
**TOTALE SCORE**		

Legend - Quality of the study	
0-4 points	Low
5-10 points	Moderate
11-16 points	High

Table S4. Result of the quality assessment process. *Note: Quality scores in Table S4 reflect multiple reporting domains captured by criteria Q1–Q9 (study design descriptors, sampling and analytical reporting, and QA/QC information), rather than the analytical protocol alone. Consequently, studies that rely on similar measurement programmes or protocols may still receive different total scores due to differences in study-specific characteristics and completeness of reporting across these domains.*

Reference	Q1	Q2	Q3	Q4	Q5	Q6	Q7	Q8	Q9	EVALUATION	
**Reference**	2	2	0	1	3	0	1	0	1	Moderate	10
(Cena *et al.*, 2014)	2	3	1	1	4	1	1	1	0	High	14
(Decharat, 2015)	2	3	1	0	4	0	1	0	1	High	12
(Ghobakhloo *et al.*, 2024)	2	3	1	1	3	1	1	1	1	High	14
(Wang *et al.*, 2022)	2	3	1	1	4	0	1	1	0	High	13
(Shaw *et al.*, 2020)	2	2	1	1	3	1	2	1	1	High	14
(Spinazzè *et al.*, 2022)	2	2	0	0	3	0	2	0	1	Moderate	10
(Ščančar *et al.*, 2015)	2	2	0	1	3	0	2	0	1	High	11
(Mariem Nafti Radhouane Chakroun and Nouaïgui, 2017)	2	2	0	1	0	1	1	0	1	Moderate	8
(Pourbakhshi *et al.*, 2021)	2	2	0	1	2	0	2	1	1	High	11
(Bau *et al.*, 2020)	2	2	1	0	2	0	2	0	1	Moderate	10
(Newton *et al.*, 2023)	2	2	0	0	2	0	2	1	0	Moderate	9
(Berlinger and Harper, 2018)	2	1	1	1	1	0	0	0	0	Moderate	6
(Galarneau, 2022)	2	2	0	0	1	0	2	1	1	Moderate	9
(Kato *et al.*, 2022)	2	3	0	0	0	0	0	0	0	Moderate	5
(Sarwar *et al.*, 2023)	2	3	0	1	3	1	2	1	0	High	13
(Khalili and Nasrabadi, 2023)	2	3	0	1	4	0	2	0	0	High	12
(Onat *et al.*, 2020)	2	3	1	1	4	0	1	1	0	High	13
(Yang *et al.*, 2018a)	2	3	1	0	3	1	2	1	1	High	14
(Soltanpour *et al.*, 2023)	2	3	0	0	3	0	1	0	0	Moderate	9
(Galvão *et al.*, 2014)	2	3	0	0	3	0	2	0	1	High	11
(Wu *et al.*, 2021)	2	3	1	1	4	0	2	0	0	High	13
(Järvelä *et al.*, 2016)	2	3	1	0	3	1	0	1	0	High	11
(Mehrifar *et al.*, 2020)	2	3	1	1	4	0	2	0	0	High	13
(Stanislawska *et al.*, 2017)	2	3	1	0	3	0	2	0	1	High	12
(Wu and Liu, 2014)	2	2	0	1	3	0	1	0	1	Moderate	10
(Gu *et al.*, 2023)	2	3	1	1	4	1	0	1	0	High	13
(Viegas *et al.*, 2022)	2	3	0	0	4	0	1	0	0	Moderate	10
(Graff *et al.*, 2017)	2	3	1	1	4	0	2	0	1	High	14
(Pesch *et al.*, 2018)	2	3	1	0	3	0	2	0	1	High	12
(Engelsman *et al.*, 2019)	2	3	1	0	3	0	2	0	1	High	12
(Julander *et al.*, 2014)	2	3	1	0	4	1	2	0	0	High	13
(Khoshakhlagh *et al.*, 2023)	2	3	1	1	4	0	2	1	0	High	14
(Sepahi Zoeram *et al.*, 2022)	2	3	0	0	3	0	1	0	0	Moderate	9
(Ogundele *et al.*, 2017)	2	3	1	1	4	0	2	1	1	High	15
(Jiang *et al.*, 2024)	2	3	1	1	3	0	1	0	0	High	11
(Lehnert *et al.*, 2022)	2	3	1	0	4	1	2	0	0	High	13
(Lau *et al.*, 2014)	2	3	1	0	4	0	2	0	0	High	12
(Newton *et al.*, 2021b)	2	3	1	1	4	0	2	1	0	High	14

(Hamzah *et al.*, 2016)	2	3	1	0	3	1	2	1	1	High	14
(Gerding *et al.*, 2021)	2	3	1	1	1	1	2	1	0	High	12
(Zendehdel *et al.*, 2019)	2	3	1	1	3	1	2	0	1	High	14
(Vincent *et al.*, 2015)	2	3	1	0	3	0	2	1	0	High	12
(Martin *et al.*, 2015)	2	3	1	0	4	1	0	0	0	High	11
(Oddone *et al.*, 2022)	2	3	0	0	4	1	2	1	1	High	14
(Keyter *et al.*, 2019)	2	3	1	0	3	0	0	0	0	Moderate	9
(Laitinen *et al.*, 2017)	2	2	0	0	3	0	1	0	0	Moderate	8
(Huang *et al.*, 2016)	2	3	0	0	4	0	1	0	0	Moderate	10
(Miettinen *et al.*, 2016)	2	3	1	0	4	1	2	1	0	High	14
(Lehnert *et al.*, 2014)	2	3	1	0	2	0	0	1	0	Moderate	9
(Chen *et al.*, 2014)	2	3	1	1	4	1	1	0	0	High	13
(Oginawati *et al.*, 2021)	2	3	1	0	2	0	0	0	0	Moderate	8
(Hamid *et al.*, 2023)	2	3	1	0	4	0	2	1	0	High	13
(Nurul *et al.*, 2016)	2	3	1	0	4	0	2	0	0	High	12
(Arsal Yıldırım *et al.*, 2020)	2	3	1	1	3	1	2	0	1	High	14
(Kettelarij *et al.*, 2016)	2	3	1	0	1	0	1	0	0	Moderate	8
(Sikder *et al.*, 2017)	2	3	1	0	3	1	2	0	1	High	13
(Dueck *et al.*, 2021)	2	3	1	1	2	0	2	0	1	High	12
(Cena *et al.*, 2015)	2	3	1	0	4	0	2	0	1	High	13
(Eriksen Hammer *et al.*, 2022)	2	3	1	1	3	0	2	1	0	High	13
(Kamaludin *et al.*, 2020)	2	3	1	0	3	1	1	0	0	High	11
(Kalteh *et al.*, 2020)	2	3	1	1	3	0	0	1	0	High	11
(Bennett *et al.*, 2016)	2	3	1	0	4	0	2	1	0	High	13
(Yang *et al.*, 2018b)	2	3	0	0	4	1	2	1	0	High	13
(Lee *et al.*, 2023)	2	3	1	0	4	0	1	1	1	High	13
(Hanser *et al.*, 2022)	2	3	0	1	1	0	0	0	0	Moderate	7
(Sakebayeva *et al.*, 2018)	2	3	1	0	4	1	1	1	1	High	14
(Gravel *et al.*, 2023)	2	3	1	0	4	0	1	0	1	High	12
(Newton *et al.*, 2021a)	2	2	1	0	4	0	1	0	1	High	11
(Park *et al.*, 2017)	2	3	1	0	4	0	1	1	0	High	12
(Cate *et al.*, 2014)	2	3	1	0	3	0	2	0	0	High	11
(López *et al.*, 2023)	2	3	1	0	4	1	1	1	1	High	14
(Vattanasit *et al.*, 2021)	2	2	0	1	3	0	2	1	1	High	12
(Wang *et al.*, 2017)	2	3	1	0	4	0	2	0	0	High	12
(Mitra and Das, 2022)	2	3	1	0	4	1	1	1	1	High	14
(Jafari *et al.*, 2023)	2	1	1	1	0	0	0	0	0	Moderate	5
(Peters *et al.*, 2015)	2	1	1	1	4	1	1	0	0	High	11
(Pesch *et al.*, 2015)	2	3	1	1	4	1	1	1	1	High	15
(Pourhassan *et al.*, 2024)	2	3	0	1	3	1	1	1	0	High	12

Table S5. List of papers found suitable and reviewed in this study by year of publication.

Publication Year	n	%	References
2014	8	10,1%	(Wu and Liu, 2014; Cena *et al.*, 2014; Lau *et al.*, 2014; Cate *et al.*, 2014; Galvão *et al.*, 2014; Lehnert *et al.*, 2014; Chen *et al.*, 2014; Julander *et al.*, 2014)
2015	7	8,9%	(Cena *et al.*, 2015; Decharat, 2015; Peters *et al.*, 2015; Vincent *et al.*, 2015; Pesch *et al.*, 2015; Ščančar *et al.*, 2015; Martin *et al.*, 2015)
2016	7	8,9%	(Bennett *et al.*, 2016; Huang *et al.*, 2016; Hamzah *et al.*, 2016; Järvelä *et al.*, 2016; Miettinen *et al.*, 2016; Nurul *et al.*, 2016; Kettelarij *et al.*, 2016)
2017	8	10,1%	(Graff *et al.*, 2017; Mariem Nafti Radhouane Chakroun and Nouaïgui, 2017; Park *et al.*, 2017; Stanislawska *et al.*, 2017; Wang *et al.*, 2017; Sikder *et al.*, 2017; Ogundele *et al.*, 2017; Laitinen *et al.*, 2017)
2018	5	6,3%	(Sakebayeva *et al.*, 2018; Berlinger and Harper, 2018; Pesch *et al.*, 2018; Yang *et al.*, 2018b, 2018a)
2019	3	3,8%	(Zendehdel *et al.*, 2019; Keyter *et al.*, 2019; Engelsman *et al.*, 2019)
2020	7	8,9%	(Bau *et al.*, 2020; Kamaludin *et al.*, 2020; Mehrifar *et al.*, 2020; Onat *et al.*, 2020; Shaw *et al.*, 2020; Kalteh *et al.*, 2020; Arsal Yıldırım *et al.*, 2020)
2021	8	10,1%	(Newton *et al.*, 2021b, 2021a; Pourbakhshi *et al.*, 2021; Vattanasit *et al.*, 2021; Wu *et al.*, 2021; Oginawati *et al.*, 2021; Gerding *et al.*, 2021; Dueck *et al.*, 2021)
2022	11	13,9%	(Kato *et al.*, 2022; Mitra and Das, 2022; Wang *et al.*, 2022; Galarneau, 2022; Lehnert *et al.*, 2022; Viegas *et al.*, 2022; Sepahi Zoeram *et al.*, 2022; Oddone *et al.*, 2022; Eriksen Hammer *et al.*, 2022; Hanser *et al.*, 2022; Spinazzè *et al.*, 2022)
2023	13	16,4%	(Gu *et al.*, 2023; Khoshakhlagh *et al.*, 2023; Hamid *et al.*, 2023; Khalili and Nasrabadi, 2023; Gravel *et al.*, 2023; Lee *et al.*, 2023; van Ree *et al.*, 2023; López *et al.*, 2023; Soltanpour *et al.*, 2023; Jafari *et al.*, 2023; Newton *et al.*, 2023; Sarwar *et al.*, 2023; Pourhassan *et al.*, 2024)
2024	2	2,6%	(Ghobakhloo *et al.*, 2024; Jiang *et al.*, 2024)

Table S6. List of papers found suitable and reviewed in this study by area of the study.

**Country/Nation**	**n**	**%**	**References**
Australia	1	1,3%	(Engelsman *et al.*, 2019)
Bangladesh	1	1,3%	(Sikder *et al.*, 2017)
Brazil	1	1,3%	(Galvão *et al.*, 2014)
Canada	5	6,4%	(Peters *et al.*, 2015; Shaw *et al.*, 2020; Dueck *et al.*, 2021; Galarneau, 2022; Gravel *et al.*, 2023)
China	3	3,8%	(Chen *et al.*, 2014; Huang *et al.*, 2016; Gu *et al.*, 2023)
Finland	2	2,5%	(Järvelä *et al.*, 2016; Laitinen *et al.*, 2017)
France	2	2,5%	(Vincent *et al.*, 2015; Hanser *et al.*, 2022)
Germany	5	6,4%	(Lehnert *et al.*, 2014, 2022; Pesch *et al.*, 2015, 2018; Gerding *et al.*, 2021)
Hong Kong	1	1,3%	(Lau *et al.*, 2014)
Indonesia	1	1,3%	(Oginawati *et al.*, 2021)
Iran	8	10,1%	(Mehrifar *et al.*, 2020; Kalteh *et al.*, 2020; Sepahi Zoeram *et al.*, 2022; Khoshakhlagh *et al.*, 2023; Soltanpour *et al.*, 2023; Jafari *et al.*, 2023; Ghobakhloo *et al.*, 2024; Pourhassan *et al.*, 2024)
Italy	2	2,6%	(Oddone *et al.*, 2022; Spinazzè *et al.*, 2022)
Kazakhstan	1	1,3%	(Sakebayeva *et al.*, 2018)
Malaysia	3	3,8%	(Hamzah *et al.*, 2016; Nurul *et al.*, 2016; Kamaludin *et al.*, 2020)
Nigeria	1	1,3%	(Ogundele *et al.*, 2017)
Norway	2	2,5%	(Berlinger and Harper, 2018; Eriksen Hammer *et al.*, 2022)
Pakistan	2	2,5%	(Hamid *et al.*, 2023; Sarwar *et al.*, 2023)
Poland	1	1,3%	(Stanislawska *et al.*, 2017)
South Africa	1	1,3%	(van Ree *et al.*, 2023)
Spain	1	1,3%	(López *et al.*, 2023)
Sweden	4	5,1%	(Julander *et al.*, 2014; Kettelarij *et al.*, 2016; Graff *et al.*, 2017; Jiang *et al.*, 2024)
Taiwan	3	3,8%	(Wu and Liu, 2014; Yang *et al.*, 2018b, 2018a)
Thailand	2	2,5%	(Decharat, 2015; Vattanasit *et al.*, 2021)
Tunisia	1	1,3%	(Mariem Nafti Radhouane Chakroun and Nouaïgui, 2017)
Turkey	2	2,5%	(Onat *et al.*, 2020; Arsal Yıldırım *et al.*, 2020)
USA	6	7,6%	(Cena *et al.*, 2015; Martin *et al.*, 2015; Newton *et al.*, 2021b, 2021a, 2023; Wu *et al.*, 2021)
West Bengal	1	1,3%	(Mitra and Das, 2022)
Various (multi-center)	1	1,3%	(Viegas *et al.*, 2022)
Not available, not reported	15	18,9%	(Cena *et al.*, 2014; Cate *et al.*, 2014; Ščančar *et al.*, 2015; Bennett *et al.*, 2016; Miettinen *et al.*, 2016; Park *et al.*, 2017; Wang *et al.*, 2017, 2022; Zendehdel *et al.*, 2019; Keyter *et al.*, 2019; Bau *et al.*, 2020; Pourbakhshi *et al.*, 2021; Kato *et al.*, 2022; Khalili and Nasrabadi, 2023; Lee *et al.*, 2023)

Table S7. List of papers found suitable and reviewed in this study occupational sector investigated.

Occupational sector	n	References
Additive manufacturing	3	(Graff *et al.*, 2017; Oddone *et al.*, 2022; van Ree *et al.*, 2023)
Automotive	3	(Vattanasit *et al.*, 2021; Sepahi Zoeram *et al.*, 2022; Khoshakhlagh *et al.*, 2023)
Cement industry	2	(Kamaludin *et al.*, 2020; Jafari *et al.*, 2023)
Electroplating	1	(Chen *et al.*, 2014)
E-waste recycling	3	(Lau *et al.*, 2014; Julander *et al.*, 2014; Gravel *et al.*, 2023)
Leather, Tanning	1	(Sarwar *et al.*, 2023)
Metal industries	8	(Vincent *et al.*, 2015; Järvelä *et al.*, 2016; Ogundele *et al.*, 2017; Zendehdel *et al.*, 2019; Keyter *et al.*, 2019; Onat *et al.*, 2020; Viegas *et al.*, 2022; Jiang *et al.*, 2024)
Painting	1	(Bennett *et al.*, 2016)
Pipeline construction	2	(Yang *et al.*, 2018b, 2018a)
Printing	1	(Decharat, 2015)
Shipyards	2	(Wang *et al.*, 2022; López *et al.*, 2023)

Table S8. Study-level sampling and analytical method details (quality criteria Q5-Q9). *Abbreviations/notes: P = personal (breathing-zone) sampling; Stat. = stationary/area sampling. If both are indicated for the same study record, both sampling approaches were used and/or reported. Fraction/size convention: Inh = inhalable; Resp = respirable; Thor = thoracic; TSP = total suspended particulate; $PM_{2.5}/PM_{10}$ = particulate matter with aerodynamic diameter ≤2.5/10 µm. NR = not reported; LOD = limit of detection; LOQ = limit of quantification. Common samplers/materials (when used): IOM = Institute of Occupational Medicine inhalable sampler; CFC = closed-face cassette; NRD = nanoparticle respiratory deposition sampler; MCE = mixed cellulose ester; PVC = polyvinyl chloride; PTFE = polytetrafluoroethylene. Analytical techniques (examples): IC = ion chromatography; HPLC–ICP-MS = high-performance liquid chromatography coupled to ICP-MS; ICP-MS/ICP-OES(ICP-AES) = inductively coupled plasma mass spectrometry/optical emission spectrometry; ETAAS/GFAAS = electrothermal/graphite furnace atomic absorption spectrometry; FP-XRF = field-portable X-ray fluorescence; SEM-EDS/TEM-EDS = scanning/transmission electron microscopy with energy-dispersive spectroscopy; XPS = X-ray photoelectron spectroscopy. "Stat." field: where used as an internal label in the study record, it indicates the type of summary statistic(s) used in the original paper to report exposure results (e.g., AM, GM, median, percentiles, range). When not populated, the statistic was not clearly reported/extracted in that source. The last column summarises key limitations/challenges (author-reported and/or coded) to support the thematic synthesis of methodological issues.*

ID	Reference	Cr species	Sampling (summary of methods)	Analysis (summary of methods and principal results)	Key limitations/challenges
1	Cena et al. 2014	Both (Cr(VI) + total Cr/metals)	Sampling: Not reported	Instrument/notes: Inductively coupled plasma mass spectrometry (ICP-MS) and ion chromatography (IC).	Sampling approach/parameters incompletely reported; Sampling duration not consistently reported; Protocol/standard not explicitly referenced in the paper
			Fraction: Respirable	The eight screens are digested together and analyzed by inductively coupled plasma mass spectroscopy (ICP/MS) for total Cr, Ni, and Mn and by ion chromatography (IC) for Cr(VI).	
			Filter/substrate: The diffusion media consists of eight 25-mm hydrophilic nylon mesh screens with 11-µm pore size and 6% porosity (model NY1102500, Millipore Inc., Billerica, Mass.).	LOD: Mn: 1.3 µg	
			Flow: 2.5 l/min	Ni: 0.4 µg	
				Cr: 1.1 µg	
				Cr(VI): 0.4 µg; LOQ: Mn: 4.43 µg	
				Ni: 1.14 µg	
				Cr: 3.33 µg	
				Cr(VI): 1.42 µg	
2	Decharat 2015	Total Cr/metals measured	Sampling: P	Analytical method: Air: NIOSH 7024	No Cr(VI) speciation (total Cr/total metals only); Sampling approach/parameters incompletely reported; LOD/LOQ not reported
			Stat.	Blood Serum: tthis method of serum chromium determination was modified from that ofRandall and Gibson, 1987	
			Fraction: Not reported	Instrument/notes: Air: air sample filters and field blanks were subjected to slow wet acid digestion in accordance with the NIOSH standard analytical method 7024. Each sample solution was diluted with 0.1 M nitric acid to 10 mL in a volumetric flask prior to chemical analysis. The concentrations of Cr in the digested breathing zone air were determined using FAAS (furnace atomic absorption spectrophotometer) following the NIOSH meth-	

				od 7024.	
			Method/sampler: Air sampling: NIOSH 7024/1994	Blood Serum: the serum sample is added by Mg(NO3)3. The sample was dried by lyophilization and then ashed and dissolved in 0.1 HCl.	
			Filter/substrate: Air sampling: mixed cellulose ester membrane	Urine: aliquots of 10 μL of the diluted urine samples were introduced directly into a pyrolytically coated graphite furnace tube and, with an equal volume of 10 μL matrix modifier mixture (0.6% palladium nitrate and 0.15% m/V magnesium nitrate in 0.01 M nitric acid), were automatically injected sequentially. The concentrations of Cr were obtained directly from the calibration graphs after automatic correction of the absorbance of the signal from appropriate reagent blanks. Creatinine levels were analyzed in all spot urine samples using the alkaline picrate method, which was based on a modified Jaffe reaction.	
			Flow: Air sampling: 2 l/min		
			Duration: Air sampling: 8h		
3	Ghobakhloo et al. 2024	Total Cr/metals measured	Sampling: P	Instrument/notes: The mass concentration for PM2.5 was then determined by dividing the difference between the after and before weight of the glass fiber filter paper by the total volume of the air sample.	No Cr(VI) speciation (total Cr/total metals only); Sampling approach/parameters incompletely reported
			Stat.	In order to analyze HMs concentrations, 75 membrane filters used by the workers were digested using a mixture of nitric acid (HNO3) and perchloric acid (HClO4) in a ratio of 1:3 on a hot plate at a temperature of 140°C. The digestion process continued until white fumes were observed. The residues were dissolved in 0.1 M hydrochloric acid (HCl) and filtered through the Whatman filter paper no. 42. The resulting solution was then adjusted to a final volume of 15 mL using deionized water (double distilled). Concentrations of Cd, Pb, As, Cr, Cu, Ni, Co, Zn, Fe, and Mn were detected in each sample using an Inductively Coupled Plasma-Mass Spectrometry (ICP-MS; Agilent 7800 Quadrupole).	
			Fraction: $PM_{2.5}$	LOD: Not reported; LOQ: LOQ (calculated as ten times the standard deviation of ten pure solvent blank samples):	
			Filter/substrate: MCE membrane filter (37-mm and pore size 0.45 µm Pall Corp., NY, USA)	Cr: 0.093 µg/l	
			Flow: 2 l/min	As: 0.15 µg/l	
			Duration: 70-90 min.	Cu: 0.3 µg/l	
				Ni: 0.26 µg/l	
				Cd: 0.051 µg/l	
				Pb: 2 µg/l	
				Co: 0.5 µg/l	
				Zn: 0.3 µg/l	
				Fe: 0.16 µg/l	

				Mn: 0.17 µg/l	
4	Wang et al. 2022	Total Cr/metals measured	Sampling: P	Analytical method: Chamber experiment for determining metal fume emission rates: NIOSH 7300	No Cr(VI) speciation (total Cr/total metals only); Sampling approach/parameters incompletely reported
			Stat.	Determining metal fume concentrations in welding simulation experiment: NIOSH 7303	
			Fraction: Inhalable	Field monitoring on metal fume exposure concentrations of workers in a shipyard factory: NIOSH 7303	
			Method/sampler: Japanese Industrial Standards Z 3930	Instrument/notes: Chamber experiment for determining metal fume emission rates: all collected samples were analyzed for selected metals by Inductively Coupled Plasma Atomic Emission Spectroscopy (ICP-AES) analysis following NIOSH Method 7300.	
			Flow: Determining metal fume concentrations in welding simulation experiment: 2 l/min	Determining metal fume concentrations in welding simulation experiment: Inductively Coupled Plasma Mass Spectrometry (ICP-MS)	
			Field monitoring on metal fume exposure concentrations of workers in a shipyard factory: 2 l/min	Field monitoring on metal fume exposure concentrations of workers in a shipyard factory: Inductively Coupled Plasma Mass Spectrometry (ICP-MS)	
			Duration: Chamber experiment for determining metal fume emission rates: the sampling time for each testing condition was set at 300 s, including 30 s for arcing time, and the subsequent 240 s for the arc of period.	LOD: Not reported; LOQ: Determining metal fume concentrations in welding simulation experiment:	
			Determining metal fume concentrations in welding simulation experiment: the welding time was 30 min.	LOQ for each metal element was 0.003 µg	
			Field monitoring on metal fume exposure concentrations of workers in a shipyard factory: 8h	Field monitoring on metal fume exposure concentrations of workers in a shipyard factory:	
				LOQ for each metal element was 0.003 µg	
5	Shaw et al. 2020	Both (Cr(VI) + total Cr/metals)	Sampling: Stat.	Analytical method: Hexavalent chromium: NIOSH 7605 (the inhalable dust samples were analysed in the same way, except that the complete sampling assembly was weighed)	LOD/LOQ not reported
			Fraction: Inhalable	Total dust samples - gravimetric: NIOSH 0500 for the total dust samples and NIOSH Method 0500 modified for inhalable samplers	
			Filter/substrate: Inhalable dust (ID): 25-mm diameter 5 µm pore size PVC filter	Instrument/notes: The total dust and inhalable dust samples were first analysed gravimetrically by weighing on a Cahn-44 microbalance, using NIOSH Method 0500 (NIOSH, 2003a) for the total dust samples and NIOSH Method 0500 modified for inhalable samplers.	
			Flow: Total dust (TD): 2 l/min	Hexavalent chromium: anionexchange liquid chromatography with post-column colorimetric detection using diphenylcarbazide.	
			Inhalable dust (ID): 2 l/min		
			Duration: The sampling duration ranged from 60 min to 461 min		

			Sample duration:		
			steel passivation: 334 min		
			welding: 60 min		
			electroplater A: 443 min		
			electroplater B: 461 min		
6	Spinazzè et al. 2022	Both (Cr(VI) + total Cr/metals)	Sampling: P	Prep: Extraction (2 cases)	Sampling duration not consistently reported
			Stat.	Analytical method: NIOSH 7600 modified ISO 17075	
			Fraction: Inhalable	Instrument/notes: Chromatographic Separation and ICP-MS Detection/ LC-ICP-MS(Quality Assurance and Quality Control of the Developed Protocol)	
			Method/sampler: Air sampling; NIOSH 7600-7605	LOD: Cr: 3 ug/Kg; LOQ: Cr: 0.0039 ug/m3	
			Filter/substrate: PVC filters (PALL-GLA-5000 low-ash PVC membranes; 37 mm in diameter; porosity 5 μm)		
			Flow: 2 L/min		
7	Ščančar et al. 2015	Both (Cr(VI) + total Cr/metals)	Sampling: P	Prep: For total Metals: digested in Teflon autoclaves with a mixture of 2 mL aqua regia and 0.2 mL hydrofluoric acid; Alkaline extraction procedure	Sampling approach/parameters incompletely reported; Flow rate not consistently reported; Protocol/standard not explicitly referenced in the paper
			Fraction: Inhalable	Instrument/notes: HPLC–ICP-MS	
			Filter/substrate: (PVC) mem_x005F_x0002_brane filters (Millipore Corp., Billerica, MA, USA)	LOD: 0.02 ng Cr mL(-1)	
			Duration: 60 min.	molybdate (0.1 ng Mo mL_x005F_x0002_1)	
				tungstate (0.1 ng W mL_x005F_x0002_1); LOQ: Not reported	
8	Mariem Nafti et al. 2017	Both (Cr(VI) + total Cr/metals)	Sampling: P	Prep: Solid-phase extracion in order tu remove the unretained Cr(III)	Protocol/standard not explicitly referenced in the paper
			Stat.	Instrument/notes: Solid phase extracion and ETAAS analisys	
			Fraction: Inhalable	LOD: 0.1 ug/L; LOQ: 0.4ug/L	
			Filter/substrate: 37 mm cassettes containing PVC FILTERS		
			Flow: 2 l/min		
			Duration: 8 h		
9	Pourbakhshi et al. 2021	Both (Cr(VI) + total Cr/metals)	Sampling: P	Instrument/notes: SFOD	Flow rate not consistently reported; Sampling duration not consistently reported
			Fraction: Inhalable	LOD: 0.5/30 ug/l; LOQ: 0.05/0.21 ug /L	
			Method/sampler: Air samplig: NIOSH 7600		
10	Bau et al. 2020	Both (Cr(VI) + total Cr/metals)	Sampling: P	Prep: Chemical analisys: Microwave Digestion single reaction chamber (SRC)	Sampling approach/parameters incompletely reported; Flow

					rate not consistently reported; Sampling duration not consistently reported
			Stat.	Analytical method: ISO 15202-2 (digestion)	
			Fraction: Inhalable	ISO 15202-3 (ICP-AES)	
			Respirable	ISO 30011(ICP-MS)	
			Filter/substrate: PVC filters (Zeflon) with a pore size of 5um 27 mm (inhalable), 37 mm (respiratory fractions)	Instrument/notes: ICP-MS	
				LOD: Not reported; LOQ: 0.098 ug/m3	
11	Newton et al. 2023	Both (Cr(VI) + total Cr/metals)	Sampling: P	Prep: Gravimetric analysis: HSE metohd MDHS 14/4 2014	Sampling approach/parameters incompletely reported; Flow rate not consistently reported; Sampling duration not consistently reported
			Fraction: Inhalable	Instrument/notes: NITON portable XRF model XL3t series 600 (Thermo Fisher scientific)+ Trasparent Mylan film. the IOM filters were placed on the Mylan film for analysis so that 8-mm x rays beem would pass in the center of the filters.	
			Filter/substrate: MCE membrane filter (25 mm, 0.8 um pore size)	LOD: 0.09/0.6 ug/cm3; LOQ: Not reported	
12	Berlinger et al. 2018	Both (Cr(VI) + total Cr/metals)	Sampling: Stat.	Prep: Gravimetric analysis	Flow rate not consistently reported; LOD/LOQ not reported
			Fraction: Inhalable	Analytical method: Iso: 15202: dissolve soluble metal and metalloid compound with water complying with the requirements for ISO 3696	
			Duration: 45 min.	Instrument/notes: ICP- AES o ICP-MS	
14	Galarneau 2022	Total Cr/metals measured	Sampling: Not reported	Not reported	No Cr(VI) speciation (total Cr/total metals only); Sampling approach/parameters incompletely reported; Flow rate not consistently reported; Sampling duration not consistently reported
			Fraction: Not reported		
18	Kato et al. 2022	Total Cr/metals measured	Sampling: P	Prep: The sample filters were cut into approximately 4 × 4 mm pieces and fixed onto SEM specimen mounts with a carbon conductive tape. The fumes collected on the Isopore filters were observed through field emission SEM (FE-SEM; JSM-7900F, JEOL, Japan) at an accelerating voltage of 1.0 kV and then analyzed through EDS (X-Max 150; Oxford Instruments, UK) under an accelerating voltage of 10 kV	No Cr(VI) speciation (total Cr/total metals only); Sampling approach/parameters incompletely reported; Flow rate not consistently reported; Sampling duration not consistently reported
			Fraction: Inhalable	Analytical method: NIOSH 7304	
				Instrument/notes: Bulk anal_x005F_x0002_ysis of the metals was conducted through ICP-AES (Optima 7300 DV;	
				PerkinElmer, USA). ICP-AES was operated in the axial-view mode	

				LOD: Not reported; LOQ: Cr: 6.11 ppb	
19	Sarwar et al. 2023	Total Cr/metals measured	Sampling: Not reported	Not reported	No Cr(VI) speciation (total Cr/total metals only); Sampling approach/parameters incompletely reported; Flow rate not consistently reported; Sampling duration not consistently reported
			Fraction: Inhalable		
20	Khalili et al. 2023	Total Cr/metals measured	Sampling: P	Prep: Digested in a 4 to 1 solution of nitric acid (HNO3) and perchloric acid (HClO4)	No Cr(VI) speciation (total Cr/total metals only); LOD/LOQ not reported
			Fraction: Inhalable	Analytical method: NIOSH 7300 2003	
			Method/sampler: NIOSH 1501	1501 2003	
			Filter/substrate: Cellulose ester membrane flter (0.8-µm pore size)	Instrument/notes: Inductively coupled plasma atomic emission spectroscopy (ICP-AES)	
			Flow: 1-4 l/min		
			Duration: 8–10 am, 11		
			am–1 pm, 3–5 pm		
21	Onat et al. 2020	Total Cr/metals measured	Sampling: P	Prep: Gravimetric analysis, microwave system (1 mL HCl (30%), and 0.5 mL hydrogen fluoride, 35 min)	No Cr(VI) speciation (total Cr/total metals only); LOD/LOQ not reported
			Fraction: Inhalable	Instrument/notes: GF-AAS 600 graphite atomic absorption spectrophotometer	
			Respirable		
			Filter/substrate: 25-mm polytetrafluoroethylene filter at the appropriate stage with par_x005F_x0002_ticles < 0.25 µm collected on a 37-mm after-filter		
			Flow: 9 L/min		
			Duration: 8 h during a work shift		
22	Yang et al. 2018	Total Cr/metals measured	Sampling: Not reported	Analytical method: NIOSH analytical method 7301	No Cr(VI) speciation (total Cr/total metals only); Sampling approach/parameters incompletely reported; LOD/LOQ not reported
			Fraction: Not reported	Instrument/notes: ICP-MS, Agilent 7500ce, WA, USA	
			Filter/substrate: 37 mm mixed cellulose ester mem_x005F_x0002_branes (0.8 µm pore size)		
			Flow: 2 l/min		
			Duration: 6 h		
23	Soltanpour et al. 2023	Both (Cr(VI) + total Cr/metals)	Sampling: P	Prep: Acidic digestion method using HCl and HNO3; he extraction of metal fumes (Cr, Ni and Fe) was based on acidic digestion method using HCl and HNO3. To prepare the samples, the following steps were performed in order: (1) The flters were gently transferred to a breaker using	Sampling approach/parameters incompletely reported

				pliers; (2) 3 ml of concentrated HCl was added to the flter and placed on the watch glass and heated to 140°C until 0.5 cc of the solution remained; (3) 3 ml of concentrated HCl was added to the flter and the watch glass placed on it and heated to 140°C until 0.5 cc of the solution remained; (5) Then 3 ml of concentrated nitric acid was added and step 3 was repeated twice, except that in the last step, 1 ml of the remaining solution was removed from the fame;	
			Fraction: Inhalable	Analytical method: NIOSH 1994	
			Method/sampler: OSHA ID-121	Instrument/notes: Graphite Furnace	
			Filter/substrate: 25 mm MCE flter with 0.8 pore size with a closed face holder	Atomic Absorption Spectrophotometry (GFAAS)	
			Flow: 2 l/min	LOD: The Limit of Detection (LOD) for Cr, Ni, and Fe were 1.50 ppb, 0.15 ppb, and 1 ppb, respectively; LOQ: Not reported	
			Duration: 480 L minimum (min 240 minuti=4h)		
24	de Oliveira Galvão et al. 2014	Total Cr/metals measured	Sampling: P	Instrument/notes: y energy dispersive	No Cr(VI) speciation (total Cr/total metals only); Sampling duration not consistently reported; LOD/LOQ not reported; Protocol/standard not explicitly referenced in the paper
			Fraction: PM2.5	X-ray fluorescence spectrometry analysis (ED-XRF) using an EDX-700HS model	
			Filter/substrate: Polycarbonate membrane (diameter of 37 mm, pore 0.8 mm, Millipore-USA); 10 filters per site, obtaining a total of 60 filters.	(Shimadzu Corporation Analytical Instruments Division, Kyoto, Japan)	
			Flow: 1.8 lpm		
25	Wu et al. 2021	Total Cr/metals measured	Sampling: P	Prep: Gravimetric analysis	No Cr(VI) speciation (total Cr/total metals only); Sampling approach/parameters incompletely reported; Sampling duration not consistently reported
			Fraction: PM2.5	Instrument/notes: 1.5 mL microcentrifuge tube and mixed with 1 mL acid mixture (100 mL nitric acid, 10 mL hydrofluoric acid, and 1 mL 1000 ppm $AuCl_3$). The samples were then floated in a boiling water bath for 2h. Some samples were moved to SCP Science DigiPREP MS digestion block to more fully immerse the filters in the acid mixture if the samples were not well digested. Afterwards, the SCP digestion tubes were cleaned by adding 10 mL 50% v/v ultrapure nitric acid, capped and heated in the digestion block for 2h at 95 ◦C. The tubes were cooled and rinsed 3 times with deionized water. Using Teflon coated tweezers, the filters were pulled out of the microcentrifuge tube. The samples were transferred to	

				a cleaned 50 mL SCP digestion tube. Acid from 1.5 mL microcentrifuge tubes was added into the 50 mL tube, and transfer was completed by rinsing the original vessel with 1 mL of acid mixture. The samples were then heated in the digestion block, with caps on, at 95 °C for ~16h (overnight). Afterwards, they were removed from the heat, allowed to cool, and diluted to the 10 mL mark with deionized water spiked with 11.11 ppb of the trace metal standards to give a concentration of 10 ppb in the final solution. Samples were swirled to ensure even mixing. Twenty-two metals (Ag, Al, As, Ba, Be, Cd, Co, Cr, Cu, Hg, K, Mn, Mo, Ni, Pb, Sb, Se, Th, Tl, U, V, and Zn) were	
			Filter/substrate: 25-mm Teflon filter (3.0 µm porosity) (Pall Teflo®, Port Washington, NY)	then quantified using Thermo Finnigan Element 2 Inductively Coupled Plasma Sector Field Mass Spectrometer (ICP-MS).	
			Flow: 0.4 lpm.	LOD: 634.78 ± 78.94 ug/g; LOQ: Not reported	
26	Järvelä et al. 2016	Both (Cr(VI) + total Cr/metals)	Sampling: P	Prep: Gravimetric analysis	LOD/LOQ not reported; Protocol/standard not explicitly referenced in the paper
			Stat.	Instrument/notes: The structure and elemental composition of particles were analyzed using TEM-EDXA	
			Fraction: Inhalable		
			Filter/substrate: Cellulose acetate filter (AAWP, diameter 25 mm; Millipore, Bedford, MA)		
			Flow: 2.0 l min−1		
			Duration: 5.5–7.4 hr (average 6.5 hr)		
27	Mehrifar et al. 2020	Total Cr/metals measured	Sampling: P	Analytical method: NIOSH 7300 method (Cr, Ni)	No Cr(VI) speciation (total Cr/total metals only); Sampling duration not consistently reported; LOD/LOQ not reported
			Fraction: Inhalable		
			Method/sampler: NIOSH 7300 method (Cr, Ni)		
			Filter/substrate: MCE filter (25 mm, 0.8_x005F_x0002_m; SKC,USA)		
			Flow: 0.2 liters per minute		
28	Stanislawska et al. 2017	Both (Cr(VI) + total Cr/metals)	Sampling: P	Prep: The surface of the samples was sputtered with conductive material – gold (layer of 15 nm), using a vacuum sputter Quorum Technologies Ltd. Analysis of the dimensions and structure of welding dust particles and elemental composition of particles on the membrane filters surface was carried out using a scanning electron microscope (SEM). + EDS	LOD/LOQ not reported; Protocol/standard not explicitly referenced in the paper
			Stat.	Instrument/notes: Gravimetric analysis+ ICP-MS	
			Fraction: Inhalable		
			Respirable		

			Filter/substrate: Inhalable: membrane filters made of cellulose nitrate (Sartorius 11304, filter diameter 25 mm) and glass fiber filters (Wathman GF/A, filter diameter 37 mm). Respirable fraction: n membrane filters made of cellulose nitrate (Sartorius 11,304, filter diameter 37 mm). The fraction of inhalable and respirable dust was sampled by simultaneous placing of three filters in the worker's breathing zone.		
			Flow: IF: 2 l/min; RF: 2.2 l/min		
			Duration: 6-7 h		
30	Wu et al. 2014	Total Cr/metals measured	Sampling: P	Prep: Microwave digestion (800 W for 15 minutes except for urine samples, which were digested for 30 minutes)	No Cr(VI) speciation (total Cr/total metals only); Sampling approach/parameters incompletely reported; Protocol/standard not explicitly referenced in the paper
			Fraction: Inhalable	Instrument/notes: Inductively coupled plasma	
			Flow: 2 L/min	Optical emission spectrometry	
			Duration: 8 h	LOD: 0.34 µg/L for Cr; LOQ: Not reported	
31	Gu et al. 2023	Both (Cr(VI) + total Cr/metals)	Sampling: Stat.	Prep: SEM/FT-IR	Sampling approach/parameters incompletely reported; Protocol/standard not explicitly referenced in the paper
			Fraction: Inhalable	LOD: Not reported; LOQ: 0.18 µg/L	
			Filter/substrate: For short-time sam_x005F_x0002_pling, sampler equipped with an alkaline microporous		
			Flow: 3 L/min		
			Duration: 15 min		
32	Viegas et al. 2022	Both (Cr(VI) + total Cr/metals)	Sampling: P	Analytical method: OSHA ID-125G	LOD/LOQ not reported
			Fraction: Inhalable	ISO 16740	
			Respirable		
			Method/sampler: CEN-EN 481:1993		
			Filter/substrate: 25 mm PVC-filters (GLA-5000, 5 µm pore size). SKC Mini-sampler was used, loaded with a pre-weighed 13 mm MCE filter, at a flow rate of 0.75 L/min, placed under the welding visor.		
			Flow: 2 L/min		
			Duration: 8 h		
34	Graff et l. 2017	Total Cr/metals measured	Sampling: P	Instrument/notes: ICP/QMS (Thermo Fisher Scientific, Waltham, MA,	No Cr(VI) speciation (total Cr/total metals only);

				USA) for metal identities	LOD/LOQ not reported
			Stat.	Laser diffraction analysis of new and used metal powder for particle-size distribution	
			Fraction: 10 to 300 nm		
			300 nm to 10 µm		
			TSP		
			Inhalable		
			Filter/substrate: 25 mm, 0.8-µm pore mixed cellulose ester filter housed inside a closed-face polystyrene cassette		
			Flow: 2.0 (L/min)		
			Duration: 45 minutes (one preparation cycle)		
35	Pesch et al. 2018	Both (Cr(VI) + total Cr/metals)	Sampling: P	Prep: Determination of respirable Cr(VI): alkaline extraction from the filter, following a reaction with diphenylcarbazide in a strongly acidic solution, as formerly described (Pesch et al., 2015).	
			Fraction: Respirable	Instrument/notes: The concentrations of total Cr and Ni in the respirable particle fraction were determined using inductively coupled plasma mass spectrometry (ICP-MS) with Thermo Scientific XSeries 2 (Thermo Fisher Ltd, Bremen, Germany) or Perkin Elmer Elan DRC II (Waltham, Massachusetts), as formerly described (Weiss et al., 2013).	
			Filter/substrate: Determination of respirable welding fume, total chromium, and nickel: cellulose nitrate filter for respirable particles of larger particles and an additional polyurethane filter for the pre-selection quartzfibre	Determination of respirable Cr(VI): Cr(VI) concentrations were determined spectrophotometrically at 540 nm	
			Determination of respirable Cr(VI): filter in order to determine shift exposure to Cr(VI)	LOD: Determination of respirable welding fume, total chromium, and nickel: ranged from 0.36 mg m−3 to 0.48 mg m−3.; LOQ: Determination of respirable welding fume, total chromium, and nickel: ranged from 0.45 µg m−3 to 8.3 µg m−3 for Cr and from 0.28 µg m−3 to 2.9 µg m−3 for Ni	
			Flow: Determination of respirable welding fume, total chromium, and nickel: 3.5 l min−1	Determination of respirable Cr(VI): ranged from 0.37 µg m−3 to 0.43 µg m−3	
			Determination of respirable Cr(VI): 3.5 l min−1		
			Duration: Determination of respirable welding fume, total chromium, and nickel: Forty-three measurements took 4 h and seven measurements between 3 and 4 h		
36	Engelsman et al. 2019	Unclear	Sampling: Stat.	Prep: All elements reported were stripped of oxygen.	Cr species unclear/secondary estimate (interpret with caution); Sampling approach/parameters incompletely reported; Sampling duration not

					consistently reported; Protocol/standard not explicitly referenced in the paper
			Fraction: Not reported	Instrument/notes: X-ray fluorescence spectrometry and UniQuant	
			Filter/substrate: 25mm PVC membrane	LOD: 1 - 8 µg	
			Flow: 2 L/min	Specific LODs for metals can be found in the Supplementary Information (Limits of Detection for Metals Analysed). Supplementary data to this article can be found online at https://doi.org/10.1016/j.envres.2019.108745	
				LOQ: Not reported	
37	Julander et al. 2014	Total Cr/metals measured	Sampling: P	Prep: Prior to the measurements, we digested the air filters, blood, and plasma samples in acid using a Milestone ultraCLAVE II microwave digestion system (EMLS, Leutkirch, Germany) as previously described for blood samples (Kippler et al., 2009).	No Cr(VI) speciation (total Cr/total metals only); Sampling approach/parameters incompletely reported; Sampling duration not consistently reported
			Stat.	Instrument/notes: Inductively coupled plasma-mass spectrometry (ICP-MS) with a collision/reaction cell system (Agilent 7500ce, Agilent Technologies, Tokyo, Japan).	
			Fraction: Inhalable	LOD: 0.07 mg for the inhalable fraction, and 0.04 mg for OFC, calculated in accordance with ISO 15767:2009	
			Filter/substrate: Mixed cellulose esters, pore size 0.8 µm (Millipore, Bedford, MA, USA).	The limit of detection (LOD) was set to 3 times the standard deviation (SD) of the blank values. Reference materials used for quality control are presented in the supplementary material (0.0013 A1; 0.028 A2; 0.0050 A3); LOQ: Not reported	
			Flow: 2 L/min		
38	Khoshakhlagh et al. 2023	Total Cr/metals measured	Sampling: P	Prep: Each cassette filter holder also was opened and the sample filter was removed with forceps. Then, the filter was transferred to a 50-mL hot block digestion tube and digested by hydrocholeretic acid and nitric acid.	No Cr(VI) speciation (total Cr/total metals only); Sampling approach/parameters incompletely reported; LOD/LOQ not reported
			Fraction: Not reported	Instrument/notes: Atomic absorption spectrometer (SpectrAA 220FS, Varian, CA, USA).	
			Method/sampler: Metal fumes: NIOSH7303		
			Filter/substrate: Mixed cellulose esters (MCE) membrane filter produced by SKC company.		
			Flow: between 1 and 4 L per min.		
			Duration: 8:00 to 16:00 during the work shift of the workers		
39	Sepahi Zoeram et al. 2022	Total Cr/metals measured	Sampling: P	Prep: In order to prepare the filters, the acid digestion method was adopted using HNO3 and HCl. The filters were heated at 140 °C for 10 min until the whole sample turned yellow, then they were brought to the volume of 10 cc using distilled water (Safety & Health, 1994).	No Cr(VI) speciation (total Cr/total metals only); Sampling approach/parameters incompletely reported; LOD/LOQ not reported
			Fraction: Not reported	Analytical method: NIOSH Method 7303	

			Filter/substrate: Mixed cellulose ester (MCE) filter with a diameter of 37 mm and a pore size of 0.8 microns	Instrument/notes: Inductively Coupled Plasma Mass (ICP-MAS)	
			Flow: 4 lit/min		
			Duration: 120 min		
			drivers' day shift from 8 AM to 2 PM and from 4 to 8 PM		
40	Ogundele et al. 2017	Total Cr/metals measured	Sampling: Stat.	Instrument/notes: Spectro X-Lab 2000 energy-dispersive x-ray fluorescence (EDXRF) system	No Cr(VI) speciation (total Cr/total metals only); Sampling duration not consistently reported; LOD/LOQ not reported; Protocol/standard not explicitly referenced in the paper
			Fraction: PM2.5		
			PM2.5–10		
			Filter/substrate: Nuclepore polycarbonate filters of 0.4 and 8 μm pore sizes (Spurny et al., 1969a,b)		
			Flow: 16-18 L/min		
41	Jiang et al. 2023	Both (Cr(VI) + total Cr/metals)	Sampling: P	Prep: The samples were placed in glass tubes and a 5 ml solution containing sodium hydroxide (1 g/L) and sodium carbonate (1.5 g/L) was added to each tube. The filters were then extracted in an ultrasonic bath for 35 min at 40 °C. Solid residues were separated from the samples by centrifugation at 2000 rpm for 10 min. Cationic metals were removed from the samples by solid phase extraction using Dionex OnGuardTM II M columns (Thermo Fisher Scientific, GmbH, Bremen, Germany) and vacuum filtration. The liquid samples were transferred to autosampler vials. Calibration solutions in six different concentrations (30–2000 ng/ml) were diluted from a 1000 μg/ml certified stock solution (Spectrascan, Ski, Norway). The calibration solutions were diluted with the same extraction solution used for the samples.	Sampling approach/parameters incompletely reported
			Fraction: Inhalable	alkaline leaching of Cr(VI) according to ISO 15192:2006 (CEN, 2006).	
			Filter/substrate: 37 mm polyvinyl chloride filter, pore size 5 μm (Merck Millipore, Cork, Ireland).	Analytical method: Method modified from the National Institute for Occupational Safety and Health (NIOSH) (NIOSH, 2003).	
			Flow: 3.5 L/min	Instrument/notes: Ion chromatography with conductivity detection (Thermo Fisher Scientific, GmbH, Bremen, Germany, Dionex ICS-2100).	
			Duration: Full-shift work, with an average measurement time of 6.7 h.		
				LOD: The LOD was calculated using the following formula: LOD=mean value + 3 × standard deviation. The LOD was 0.08 μg/sample	
				The LOD was calculated using the following formula: LOQ = mean val-	

				ue + 10 × standard deviation. The LOQ was 0.3 µg/sample.; LOQ: Not reported	
42	Lehnert et al. 2022	Both (Cr(VI) + total Cr/metals)	Sampling: P	Instrument/notes: Mn, Ni, and total Cr: Inductively coupled plasma mass spectrometry (pectrometer of PerkinElmer LAS GmbH, Rodgau (Pitzke et al., 2019).)	Sampling duration not consistently reported; LOD/LOQ not reported
			Stat.	Cr(VI): ultraviolet–visible spectroscopy of Unicam Chromatography, type UV2-200.	
			Fraction: Respirable (total metals: Mn, Cr, Ni)		
			Inhalable (CrVI only)		
			Method/sampler: GSP 3 (respirable; EN 481)		
			GSP 10 (inhalable; CrVI only)		
			Stationary: Gravikon PM 4-2 (4 m3/h)		
			Filter/substrate: membrane filters (37 mm, 8 µm)		
			Stat: membrane filters (70 mm, 8 µm)		
			Flow: 10 l min−1		
			Stat: 4 m3 h−1		
43	Lau et al. 2014	Total Cr/metals measured	Sampling: P	Prep: Filter samples (cut into strips) were placed into Teflon tubes and digested with 4 ml mixed acid (conc. HNO3/conc. HCl=3:1) using the same microwave-assisted digestion program as the floor dust samples. The solution was cooled, filtered, and diluted to 10 ml using Milli-Q water into pretreated glass volumetric flasks.	No Cr(VI) speciation (total Cr/total metals only); Sampling approach/parameters incompletely reported; LOD/LOQ not reported; Protocol/standard not explicitly referenced in the paper
			Fraction: Not reported	Instrument/notes: Graphite Tube Atomizer (Varian Spectra AA-220Z).	
			Method/sampler: NOISH 7301		
			Filter/substrate: Quartz filter (32 mm diameter,21038, Supelco)		
			Flow: approximately 1.5 L/min		
			Duration: 24h		
44	Newton et al. 2021	Total Cr/metals measured	Sampling: P	Prep: All IOM filters and LDS substrates were digested using a strong acid microwave-assisted digestion method developed in-house to ensure total dissolution of LDS substrate. As particulate matter also deposits on the inside of the IOM cassette inlet, 2 cm × 2 cm squares of KimWipe (Thomas Scientific, Swedesboro, NJ) were used to wipe the interior of the IOM. LDS substrates were cut in half using metal-free scissors to better facilitate dissolution of the foam in the acid. Polyetherimide sampler walls were not wiped to account for	No Cr(VI) speciation (total Cr/total metals only); LOD/LOQ not reported
			Fraction: Inhalable	any possible electrostatic losses as the electrical properties of PEI do not suggest that this would be a significant source of sample loss. Briefly, IOM filters, IOM inlet wipes, and both halves of the LDS substrate were placed in separate 50 mL Teflon microwave vessels with 4 mL 70% HNO3, 2 mL 35% H2O2, and 0.35 mL 49% HF. The vessels were then	

				digested in a Mars Express Scientific Microwave (CEM Corporation, Matthews, NC) with a temperature-controlled program reaching a final temperature of 180 °C.	
			Filter/substrate: LDS: commercially manufactured foam (fiber diameter = 49.6 μm; length = 8 cm) in a sample holder engineered from heat-resistant polyetherimide (PEI) thermoplastic rods (McMaster-Carr, Elmhurst, IL).	Instrument/notes: An Agilent 7500 Series Octopole Inductively Coupled Plasma Mass Spectrometer (ICP-MS; Agilent Technologies, Santa Clara, CA) was used to analyze diluted digestant fluid. Percent recovery for all elements of interest was verified as 95 ± 5% using the welding fume CRM. Blanks, spikes, and duplicates were carried out at a rate of one in every ten samples.	
			IOM: mixed cellulose ester (MCE) filters (25 mm, 0.8 μm)		
			Flow: IOM 2 L per minute (LPM)		
			LDS 4 LPM		
			Duration: Work shift		
45	Hamzah et al. 2016	Total Cr/metals measured	Sampling: P	Prep: Wet mineral acid mixture digestion (9:1 mixture of concentrated analytical grade nitric acid and perchloric acid) on a slow heating hot plate. The acid digested matter was filtered and topped up with quartz double distilled water until 10 mL of solution was obtained. Precautions were taken to prevent extraneous contamination by usage of	No Cr(VI) speciation (total Cr/total metals only); LOD/LOQ not reported
			Fraction: Respirable	thoroughly cleaned Borosil glassware.	
			Filter/substrate: Membrane filter (0.8 μm pore size, 37 mm diameter, mixed cellulose ester)	Analytical method: gravimetric	
			Flow: 2.0 L per minute	NIOSH manual analytical methods for selected trace metals.15	
			Duration: 8h shift	Instrument/notes: Atomic	
				Absorption spectrophotometer	
46	Gerding et al. 2021	Total Cr/metals measured	Sampling: P	Prep: Samples were prepared for analysis using an open-vessel acid digestion (2 parts by volume 65% nitric acid, 1 part by volume 25% hydrochloric acid)	No Cr(VI) speciation (total Cr/total metals only); Sampling approach/parameters incompletely reported; Sampling duration not consistently reported
			Stat.	Analytical method: IFA method 7808	
			Fraction: Inhalable	Instrument/notes: Coupled plasma mass spectrometry (ICP-MS)	
			Respirable	LOD: Not reported; LOQ: Per metal dust: was determined according to the blank value method of DIN EN 32645. The LOQ was calculated as ten times the standard deviation of blank samples.	
			Method/sampler: Metal dust: IFA method 7808	Per Cr: LOQ* [μg/m3]=0.21	
			Hebisch et al. 2018	*Air monitoring LOQs depend on the air volume sampled (standard 10 l/min, 2 h) and may differ on a daily basis e.g. in case of prolonged sampling times (4 to 6 h in the present study)	
			Filter/substrate: Membrane filter (37 mm diameter, pore width 8 μm)		

			Flow: 10 l/min		
47	Zendehdel et al. 2019	Both (Cr(VI) + total Cr/metals)	Sampling: P	Prep: The amount of chromium (VI) in the samples was determined using diphenylcarbazide after acidic extraction. Filters in DCP samples, because of nickel interference, was extracted after elution with NaOH/Na2CO3 solution.	Sampling approach/parameters incompletely reported; Flow rate not consistently reported; Sampling duration not consistently reported; LOD/LOQ not reported
			Fraction: Not reported	Analytical method: Cr(VI) National Institute of Occupational Safety and Health (NIOSH) method 7600	
			Method/sampler: Cr(VI): NIOSH 7600	Ni Occupational Health and Safety Administration (OSHA) method ID-121.	
			Ni OSHA ID-121	Instrument/notes: The reaction of chromium (VI) with diphenylcarbazide was determined by a visible spectrophotometer (CECIL 2021) at 540 nm. Nickel concentration was evaluated by flame atomic absorption spectrophotometry (ANA180) after acid digestion.	
			Filter/substrate: PVC filter		
48	Vincent et al. 2015	Both (Cr(VI) + total Cr/metals)	Sampling: P	Prep: Post-column derivatization with an acidic solution of 1,5-diphenylcarbazide. A two-step extraction serves to distinguish between soluble Cr VI compounds (extraction solution at pH 8) and insoluble Cr VI compounds (alkaline extraction solution). The soluble and insoluble compounds can then be quantified compared to an external calibration curve (six	Sampling duration not consistently reported
			Stat.	concentrations (ISO, 2005).	
			Fraction: Inhalable	Instrument/notes: Ion chromatography with UV spectrophotometric detection (λ = 540 nm)	
			Method/sampler: MetroPol 084	LOD: Median limit of detection (LoD) of 24 ng; LOQ: Corresponds to a limit of quantification (LoQ) of 72 ng	
			Filter/substrate: Heat treated quartz fibre filte		
			SKC Type R-100, part number 225–1827		
			The cassette had a 4-mm sampling orifice.		
			Flow: 2 L min−1,		
49	Martin et al. 2015	Both (Cr(VI) + total Cr/metals)	Sampling: Not reported	Prep: Filters were dissolved in a mixture of high purity acids (1.0mL 16N nitric acid, 0.1mL 28N hydrofluoric acid, and 0.25mL hydrochloric acid) in Teflon bombs with a programmable microwave digestion unit (ETHOS, Milestone). Digestates were diluted to 15mL with high-purity water (18M_x005F_x0002_cm−1) and stored in pre-cleaned polyethylene bottles for 48 h.	Sampling approach/parameters incompletely reported; Flow rate not consistently reported; LOD/LOQ not reported
			Fraction: 5.6nm – 20 um	FT-IR: Toner pellets were made by mixing approximately 10mg of toner into approximately 300mg of KBr, placing this mixture into a pellet die and applying approximately 20,000 pounds per square inch (psi) pressure under a vacuum for 60 s. Similarly, micro pellets were made by mixing approximately 0.1mg PM0.1 into approximately 20mg of KBr and applying approximately 20,000 pounds psi	

				under a vacuum for approximately 60 s (5mm diameter pellet).	
			Filter/substrate: Teflon filters	Analytical method: OC/EC were analyzed using a modified NIOSH 5040 method	
			Duration: Business and non-business hours	Instrument/notes: Magnetic-sector field inductively coupled plasma mass spectroscopy (SF-ICP-MS Thermo-Finnigan 2) as described by Bello et al. [16].	
				Energy dispersive X-ray spectroscopy (EDS) (additional elemental analysis)	
				Scanning electron microscopy (SEM) and transmission electron microscopy (TEM) (PM0.1)	
				Bruker Tensor 27 using transmission IR (KBr pellet method), resolution of 4 cm−1 averaged over 32 scans	
50	Oddone et al. 2022	Total Cr/metals measured	Sampling: P	Not reported	No Cr(VI) speciation (total Cr/total metals only); Sampling approach/parameters incompletely reported; LOD/LOQ not reported
			Stat.		
			Fraction: Inhalable		
			Respirable		
			Method/sampler: Gravimetric analysis: standards UNICHIM 1998:0533		
			Gravimetric analysis - inhalable fraction: NIOSH 050034)		
			Gravimetric analysis - respirable fraction: NIOSH 060035		
			Metal analysis: NIOSH 7300		
			Filter/substrate: Mixed Cellulose Ester (MCE) Membrane Filter, with a diameter of 25 mm and porosity of 0.8 µm for gravimetric analysis		
			Polytetrafluoroethylene (PTFE) filter with a diameter of 25 mm and porosity of 0.5 µm for Sioutas, metal analysis		
			Flow: 1.4 (± 0.1) l/min for inhalable		
			2.75 (±0.1) l/min for respirable		
			9 l/min for granulometric classes		
			Duration: 3 working days or 2 with implementation of measurements		
			from 92 minutes to 480 minutes		
51	Keyter et al. 2019	Both (Cr(VI) + total Cr/metals)	Sampling: Stat.	Prep: Metal composition analysisc: inhalable samples: the filters were removed from the IOM cassettes while care was taken to remove any dust adhering to the inside walls of the cassettes in order to include it in the metal elements scan	
			Fraction: Inhalable	Particle size distribution analysis;Dynamic Light Scattering (DLS) using a Horiba LB- 550 PSD analyzer (Horiba Ltd, Kyoto, Japan)[61] to de-	

				termine the PSD range of the collected particles.	
			Thoracic	Particle size distribution analysis: Particles were removed from the filter by dipping the filter into deionized water and then removing it, repeating this process until as much as possible of the dust particles were dislodged in order to avoid bias in the results. This solution was hand shaken to mix the particles and the deionized water. A sample from the	
			Respirable	dispersed dust and deionized water mixture was added to a glass cuvette cell holder by using a pipette.[62]	
			Nano	Analytical method: Gravimetric analysis (Method: MDHS 14/4, NIOSH 0600 and NIOSH 0500).	
			Method/sampler: Methods for the Determination of Hazardous Substances: MDHS 14/4)	Metal composition analysis (Method: OSHA ID125G and NIOSH 7300).	
			Inhalable particle size fraction: MDHS 14/4	Instrument/notes: Metal composition analysis: The metal composition of the nano-size fraction was determined from the diffusion/deposition stage of the NRD sampler. For metal elements scan analysis, a Thermo Scientific Element 2 High-Resolution Inductively Coupled Plasma Mass Spectrometer (ICP-MS) (Waltham, MA)[60] was used to quantify the metal elements present. After chemical analysis, the laboratory performed blank corrections.	
			Thoracic particle size fraction: NIOSH 0600	LOD: LOD of analysis method: 0.00016 mg/m3.; LOQ: LOQ for analysis method: 0.0005 mg/m3	
			Respirable particle size fraction: NIOSH 0500		
			Filter/substrate: Eight 25-mm hydrophilic nylon mesh screens, 11 mm pore size, and 6% porosity (Zefon International Inc., Ocala, FL) for nano-size particle fraction (50% collection efficiency of the diffusion stage for 40nm particles)		
			25mm diameter, 0.8 mm pore size for mixed cellulose ester (MCE) sampling filter (SKC Inc.) for inhalable particle size fraction (50% sampling efficiency from _x005F_x0001_50–100 mm)		
			37-mm diameter, 0.8 mm pore size MCE sampling filter (SKC Inc.) for thoracic particle size fraction (50% cutpoint of 10 mm)		
			a 37-mm diameter, 0.8 mm pore size MCE sampling filter (SKC Inc.) for respirable particle size fraction (50% cut-point of 4 mm)		
			37-mm diameter, 0.8 mm pore size MCE sampling filter (SKC Inc.) for total		
			Flow: 2.5 L/min for nano		
			2.0 L/min for inhalable particle size fraction		
			1.6 L/min for thoracic particle size fraction		

			2.5 L/min for respirable particle size fraction		
			2.0 L/min for total		
			Duration: One sample was collected per day at each area of sampling. Sampling was conducted over a period of 45–108 min during gouging and 75–85 min during lancing.		
52	Laitinen et al. 2017	Both (Cr(VI) + total Cr/metals)	Sampling: Stat.	Not reported	Sampling approach/parameters incompletely reported; Sampling duration not consistently reported; LOD/LOQ not reported
			Fraction: Inhalable		
			Filter/substrate: Millipore filter (25 mm AAWP, pore size 0.8 mm, Merck Millipore)		
			Flow: 2.0 L min-1		
53	Huang et al. 2016	Total Cr/metals measured	Sampling: P	Instrument/notes: Field emission scanning electron microscope (dust particles) for discrepancy of surface morphologies	No Cr(VI) speciation (total Cr/total metals only); Sampling approach/parameters incompletely reported; Flow rate not consistently reported; LOD/LOQ not reported
			Stat.	Energy-dispersive x-ray spectrometry (EDS) and an inductively coupled plasma mass spectrometry (ICP-MS) for composition and proportion of elements contained in the dust particles	
			Fraction: Not reported	X-ray photoelectron spectroscopy (XPS) for Valence states of some elements contained in the dust particles	
			Filter/substrate: Quartz fiber filters (filter diameter at 81 mm)		
			Duration: 2.5h		
54	Miettinen et al. 2016	Total Cr/metals measured	Sampling: P	Instrument/notes: Electron microscopy	No Cr(VI) speciation (total Cr/total metals only); LOD/LOQ not reported
			Stat.	SEM (Sigma HD VP; Carl Zeiss NST, Cambridge, UK) imaging was performed at an accelerating voltage of 2 kV using two secondary electron detectors (SE2 and InLens)	
			Fraction: 9.6–420 nm	SEM–EDS from the particles and agglomerates larger than 300 nm at an accelerating voltage of 10 kV using two SDD type detectors (Thermo NS7; Thermo Scientific Inc., Madison, WI, USA)	
			5.6–560 nm	TEM (JEM-2100F; JEOL Inc., Tokyo, Japan), an accelerating voltage of 200 kV	
			Filter/substrate: Polytetrafluoroethylene (type: Teflo P/N R2PJ047; PALL Life Sciences, PALL Corp.,New York, NY, USA) filters	TEM– EDS in STEM mode using liquid nitrogen cooled detector with Si(Li) crystal (Thermo NS7; Thermo Scientific Inc.).	
			Flow: The SMPS was operated at a sample flow of 0.3 l min−1, a sheath flow of 6.0 l min−1, and a scan	Inductively coupled plasma-mass spectrometry (ICP-MS, Model 7700; Agilent Technologies, Santa Clara, CA, USA) according DIN EN ISO	

			time 135 s	17294-2 (E 29) standard.	
			Samples for the electron microscopy analyses: 0.3 l min−1		
			Duration: 26–38 min		
			Sampling: P	Prep: Sample preparation has been previously reported (Pesch et al. 2012;Hahn 2005). the filters were digested with 10 mL of a mixture of nitric acid and hydrochloric acid. This solution was heated for 2 h under reflux at 130_x005F_x0003_ C. After cooling to room temperature, the solution was diluted with 10 mL of ultrapure water before ICP-MS analysis was carried out.	No Cr(VI) speciation (total Cr/total metals only); LOD/LOQ not reported
			Fraction: Respirable	Analytical method: Weighing method as described by Hebisch et al. (2005).	
55	Lehnert et al. 2014	Total Cr/metals measured	Method/sampler: Welding fume: protocols of the WELDOX study (Pesch et al. 2012; Lehnert et al. 2012)	Instrument/notes: Inductively coupled plasma mass spectrometry (ICP-MS) with a Perkin Elmer Elan DRC II (Waltham, Massachusetts) calibrated with different multielement standard solutions. The isotopes 45Sc, 85Rb, and 165Ho were used as internal standards.	
			Filter/substrate: Cellulose nitrate filter (8 lm pore size, 37 mm diameter)		
			Polyurethane filter preselected larger particles (Moehlmann, 2006).		
			Flow: P: 3.5 L/min		
			Sta: 10 L/min		
			Duration: 3.8 h		
			Sampling: P		No Cr(VI) speciation (total Cr/total metals only); Sampling approach/parameters incompletely reported; Flow rate not consistently reported; Sampling duration not consistently reported
56	Chen et al. 2014	Total Cr/metals measured (inferred)		Analytical method: NIOSH Method 7300	
			Fraction: Not reported		
			Filter/substrate: Mixed cellulose ester filters (pore size = 0.8 µm) with a 37-mm cassette		
			Sampling: P	Instrument/notes: UV–visible spectrophotometer per Cr(VI)	LOD/LOQ not reported
			Fraction: Inhalable	Visible absorption spectrophotometry per Cr	
			Method/sampler: NIOSH 7600 (1994a, b)		
57	Oginawati et al. 2021	Both (Cr(VI) + total Cr/metals)	Filter/substrate: Polyvinyl chloride (PVC) filters		
			5.0-µm porous PVC filter diameter of 37 mm		
			Flow: 2 L/min		
			Duration: 8h		
59	Hamid et al. 2023	Total Cr/metals measured	Sampling: Stat.	Not reported	No Cr(VI) speciation (total Cr/total metals only); Sampling approach/parameters incompletely reported; Flow rate not consistently reported; LOD/LOQ not

					reported
			Fraction: TSP		
			Duration: 24h		
60	Nurul et al. 2016	Total Cr/metals measured	Sampling: P	Prep: The respirable particulate samples were subjected to wet mineral acid mixture digestion (9: 1 mixture of concentrated analytical grade nitric acid and perchloric acid) on a slow heating hot plate. The acid digested matter was filtered and made up to 10 ml using quartz double distilled water. Precautions were taken to prevent extraneous contamination by usage of thoroughly cleaned Borosil glassware	No Cr(VI) speciation (total Cr/total metals only); LOD/LOQ not reported
			Fraction: Respirable	Analytical method: NIOSH Manual Analytical Methods (NMAM) for selected trace metals	
			Filter/substrate: Filter papers	Instrument/notes: Atomic absorption spectrophotometer (AAS).	
			Flow: 2.0 litres per minute		
			Duration: 8h		
61	Arsal Yıldırım et al. 2020	Total Cr/metals measured	Sampling: Stat.	Prep: A digestion procedure was first carried out using Milestone Ethos D microwave aciddigestion system. After the PTFE filters were placed into the PTFE digestion vessels via PTFE clamps, 5 mL nitric acid (65%, w/w), 1 mL hydrochloric acid (30%, w/w), and 0.5 mL hydrofluoric acid (HF) were added. A two-step microwave digestion procedure was	No Cr(VI) speciation (total Cr/total metals only); LOD/LOQ not reported
			Fraction: PM2.5	performed after the vessels were closed (Yenisoy- Karakas et al. 2009), following which the digestion vessels were cooled to room temperature and the solution in the vessels was transferred into 50 mL tubes and diluted to 25 mL with deionized water.	
			PM1.0–2.5	Instrument/notes: Perkin Elmer Elan®DRC-e brand inductively coupled plasma mass spectrometer (ICP-MS) device.	
			PM0.5–1.0		
			PM0.25–0.50		
			PM0.25 µm		
			Filter/substrate: Polytetrafluoroethylene (PTFE) filters		
			Flow: 9 L/min		
			Duration: 7h		
62	Kettelarij et al. 2016	Total Cr/metals measured	Sampling: P	Prep: All plastic materials used for acid wipe sampling, collection of urine samples and laboratory preparation and analysis were washed in 10% HNO3 (analysis grade) for 4 h, and rinsed four times in deionized water.	No Cr(VI) speciation (total Cr/total metals only); Sampling approach/parameters incompletely reported
			Fraction: Inhalable	Instrument/notes: All samples: inductively coupled plasma mass spectrometry (Thermo Fisher Scientific iCAP™ Q ICP-MS).	
			Method/sampler: Air: EN 481	LOD: The method limit of detection (LOD) was set at 3 times the standard deviation (SD) of the blank sample matrix.	
			Flow: Air: 2 l/min	Wipe samples - LOD Cr: 0.00021 (µg/cm2)	

			Duration: 24h	Urine - LOD Cr: 0.094 (μg/l)	
				Air - LOD Cr: 0.11 (μg/l); LOQ: Not reported	
63	Sikder et al. 2017	Total Cr/metals measured	Sampling: Stat.	Instrument/notes: Laser diffraction analysis: To understand the size distribution of the particles, particle size analysis was conducted using laser diffraction particle size analyzer (Malvern Nano ZS) (Graph 1). The beakers with accumulated particles were soaked with deionized water for 24 h and then dispersed by several cycles of sonication in an ultrasound bath, to prepare the aliquot for laser diffraction analysis.	No Cr(VI) speciation (total Cr/total metals only); Flow rate not consistently reported; Sampling duration not consistently reported; LOD/LOQ not reported
			Fraction: PM2.5	SEM/EDS analysis: To directly verify the characteristics of the product of smelting, soldering, and filing of the metals that are dispersed into the air of the jewelry workshops, particles collected in the passive sampler were further analyzed with a field emission scanning electron microscope (Hitachi SU-70) equipped with an energy-dispersive X-ray spectroscopy (EDS) analyzer in the Nanomaterial Characterization Core Facility (NCC) of Virginia Commonwealth University (VCU). The particles accumulated in the samplers were prepared for electron microscopy by the following procedure. Selective samples were soaked overnight with deionized water in the 50-mL beaker, at room temperature. Then, the samples were sonicated five times for 30 min in a hot-water ultrasonic bath to achieve the optimum dispersion of the particles in the water. Very small amounts of samples were dispersed on the 0.2-μm polycarbonate filter, accomplished by dropping only 1 mL of slurry on 0.2-μm polycarbonate filters, placed over the glass fiber filters in the vacuum suction, and then the filters were dried overnight at 60 °C in the warming oven. After drying, the polycarbonate filters were transferred onto the aluminum holder with double-sided carbon tape and sputtered with carbon (C) for scanning electron microscope (SEM) analysis. And, for the analysis of non-metallic phases, particularly the carbon phases, the blank polycarbonate filters were sputtered with platinum (Pt) first and then the samples were dispersed on the filters following the same procedure and sputtered again with platinum for SEM analysis.	
			PM10	ICP analysis: To reveal the concentration of the toxic heavy metals in ultratrace levels, 0.25 g representative dust samples from corresponding workshops were digested with four acids beginning with concentrated hydrofluoric acid (49% w/w), followed by a mixture of concentrated nitric (71% w/w), concentrated perchloric acids (69% w/w) and concentrated hydrochloric acid (36% w/w), 15 h in each step, and then heated using precise programmer controlled heating in several ramping and holding cycles which takes the samples to incipient dryness. After incipient dryness is at-	

				tained, samples are brought back into solution using aqua regia. The multi-acid digested solutions were diluted and analyzed on a PerkinElmer Sciex ELAN 6000 ICP-MS. For the accuracy of metals analysis, one blank was run for every 10 samples. In-house control was run every 10 samples, and a duplicate was analyzed after every five samples.	
64	Dueck et al. 2021	Total Cr/metals measured (inferred)	Sampling: P	Prep: Filter samples were digested in a high-pressure microwave (Ultraclave, Milestone®, Leutkirch, Germany) using a mixture of 3 ml nitric acid (HNO3) and 0.1 ml of tetrafluoroboric acid (HBF4). The combination of high temperature and pressure allowed for the total dissolution of PVC filters. All acids were sub-boiled and plasticware was acid cleaned prior to use to avoid any metal contamination during sample preparation and analysis. Samples were completed to 10 ml and diluted 10-fold for field blanks and ambient air samples and 100-fold for welding fume samples using a 2% HNO3 solution.	No Cr(VI) speciation (total Cr/total metals only); Sampling approach/parameters incompletely reported; Flow rate not consistently reported
			Stat.	Instrument/notes: Inductively Coupled Plasma Mass Spectrometry (iCAP-Q Inductively Coupled Plasma Mass Spectrometry (ICP/MS) - Thermo-Finnigan®, Bremen, Germany).	
			Fraction: TSP	Filters were weighed pre- and post-sampling.	
			Method/sampler: NIOSH methods 7304	LOD was calculated as three times the standard deviation of the field blanks	
			International Standard Organization, 2011	Gravimetric analysis: 0.07 mg filter−1	
			Filter/substrate: PVC filters (5 μm; 37 mm diameter)	Metals: varied from 0.61 (Mo and V) to 670 ng filter−1 (Fe); LOQ: Not reported	
			Duration: 3-4h		
65	Cena et al. 2015	Cr(VI) measured	Sampling: P	Prep: All mild steel samples were microwave digested at 200°C in 10 mL ultrapure nitric acid and analyzed for Mn, Cr, and Ni content by inductively coupled plasma mass spectrometry.	Sampling duration not consistently reported; Protocol/standard not explicitly referenced in the paper
			Stat.	The stainless steel samples from facility A were immersed in 10 mL of extraction solution (2% sodium hydroxide, NaOH /3% Sodium carbonate, Na2CO3), placed in ultrasonic bath for 30 min, centrifuging for 15 min at 2400x g, and analyzed for Cr(VI) by ion chromatography.	
			Fraction: TSP	Instrument/notes: Cr, Mn, Ni: Inductively coupled plasma mass spectrometry	
			Respirable	Cr(VI): Ion chromatography	
			Flow: NRD sampler: 2.5 Lpm	LOD Expressed as Mass Collected in the Diffusion Stage of the NRD Sampler	
			Closed face cassettes: 2 Lpm	Total Cr: 1.1 ug	
				Cr(VI): 0.4 ug; LOQ: LOQ - Expressed as Mass Collected in the Diffusion Stage of the NRD Sampler	
				Total Cr: 3.33 ug	
				Cr(VI): 1.42 ug	

66	Hammer et al. 2022	Total Cr/metals measured	Sampling: P	Prep: *Inductively Coupled Plasma Mass Spectrometry - All PVC filters, including those with PM and field blanks, were transferred into separate vessels and added to 2 mL aqua regia (1:3, 65% HNO3, 37% HCl), 0.05 mL 40% HF (A.R. grade Sigma-Aldrich, Merck KGaA, Darmstadt, Germany), and 200 μL internal standard (2 μg/mL Rhodium). The samples were digested using a Milestone mls 1200 digestion module (Milestone, Fatebenefratelli, Italy) and diluted to 15 mL with MilliQ water.	No Cr(VI) speciation (total Cr/total metals only); Sampling approach/parameters incompletely reported
			Stat.	Instrument/notes: *SEM - Scanning Electron Microscopy: Hitachi SU6600 field emission SEM (Hitachi High-Tech, Tokyo, Japan) equipped with a Bruker EDX detector (Bruker Nano GmbH, Berlin, Germany) and a NORDIF electron backscatter diffraction (EBSD) detector (NORDIF, Trondheim, Norway). The particle morphologies were observed and imaged in secondary electron (SE) imaging mode at a working distance of 10 mm and an accelerating voltage of 15 keV. The element composition of the agglomerates/aggregates was determined by SEM-EDX point and area analysis for a selected number of particles per sample. The crystalline phase was determined with the use of the EBSD detector for 50–100 particles per sample according to the method described in Ervik et al. [20].	
			Fraction: Respirable	* Gravimetric Analysis - The mass of the PM was determined gravimetrically with a Sartorius micro model MC5 balance (Sartorius AG, Göttingen, Germany). The filters were conditioned for a minimum of two days in a room dedicated to low filter mass measurements (relative humidity 40 ± 2%, temperature 20 ± 1 °C), and discharged by a 210Po source prior to weighing. The limit of detection (LOD), calculated as three times the standard deviation of six field filter blanks, was 0.005 mg.	
			Filter/substrate: Fix	*X-ray Diffraction - The samples were analysed in a PANalytical X'Pert3 powder diffractometer, equipped with a PANalytical Empyrean X-ray tube (Malvern Panalytical, Malvern, Great Britain). In-house reference materials of magnetite and hematite were also analysed. The resulting diffractograms were examined in HighScore Plus software (Malvern Panalytical, Malvern, UK) which determined the background. The search-and-match function of the software was used to identify mineral candidates in the ICSD database. Element information from SEM and ICP-MS was used in the restriction settings to limit the number of candidates.	
			*Particulate matter formed during the laser cutting: 25 mm polyvinyl chloride (PVC) membrane filters with a pore size of 5.0 μm (Merck Millipore, Burlington, MA, USA).	*Inductively Coupled Plasma Mass Spectrometry (ICP-MS) - The quantification of Al, Cr, Cu, Fe, Mn, Ni, Pb, and Zn was performed with an Agilent 8800 QQQ ICP-MS (Agilent Technologies, Santa Clara, CA, USA). Multi-element calibration solutions (Spectrapure Standards AS, Oslo, Norway) were prepared with acid-	

				matched matrix solutions. Mild steel welding fume (MSWF-1) reference material (HSE's Science and Research Centre, Derbyshire, UK) was used for quality control.	
			*Particles for microscopy: holey carbon films (EMresolution, Sheffield, UK) were affixed to the surface of 25 mm Merck Millipore PVC membrane filters.	LOD: * Gravimetric Analysis - LOD: 0.005 mg.; LOQ: Not reported	
			Personal		
			*Personal: 37 mm FPVC filters with a pore size of 5.0 µm (Merck Millipore, Burlington, MA, USA).		
			Flow: Personal		
			Personal: 2.2 l/min.		
			Duration: Fix		
			*The filter samples for gravimetric, elemental composition, and mineral phase determination of the bulk material were collected when the cutting time was longer than 30 min.		
			*Particles for microscopy: variable sampling time between one minute to 11 min and 30 s.		
			Personal		
			Personal: 265 and 483 min.		
67	Kamaludin et al. 2020	Total Cr/metals measured	Sampling: P	Prep: The filter papers were dried in a furnace for eight hours at 100°C before they were wrapped with aluminum foil and labeled. After storage in a desiccator for 24 hours, the filter papers were weighed using a six-digit electronic microbalance (Sartorius CPA2P).	No Cr(VI) speciation (total Cr/total metals only); Sampling duration not consistently reported; LOD/LOQ not reported
			Fraction: Respirable	Analytical method: Extraction method: NIOSH Manual of Analytical Method 7300 for elements by ICP	
			Filter/substrate: Polyvinyl chloride (PVC) filter papers were used in the mineral dust sampling, with a 0.8 µm pore size and 37 mm diameter	Instrument/notes: Inductively coupled plasma mass spectrometry (ICP-MS)	
			Flow: 1.7 L min–1	The acid digestion method was performed to extract cement mineral dust from the filter papers, which was analyzed using inductively coupled plasma-mass spectrometry (ICP-MS) to determine the concentration of As and Cr. The extraction method was performed by following the National Institute of Occupational Safety and Health (NIOSH) Manual of Analytical Method 7300 for elements by ICP.	
68	Kalteh et al. 2020	Total Cr/metals measured	Sampling: P	Instrument/notes: Inductively Coupled Argon, Plasma Atomic Emission Spectroscopy (ICP-AES)	No Cr(VI) speciation (total Cr/total metals only); Sampling approach/parameters incompletely reported; Sampling duration not consistently reported; LOD/LOQ not reported

			Fraction: Not reported		
			Method/sampler: NIOSH 7300		
			Filter/substrate: Cellulose ester membrane (0.8 μm)		
			Flow: 1.5 L/min		
69	Bennett et al. 2016	Cr(VI) measured	Sampling: P	Analytical method: Gravimetric: NIOSH Method 0500	Sampling approach/parameters incompletely reported; LOD/LOQ not reported
			Stat.	Analysis: NIOSH Method 7605	
			Fraction: Not reported		
			Filter/substrate: Pre-weighed polyvinyl chloride (PVC) filters (37 mm diameter and 5.0 μm pore size)		
			Flow: 2.0 lpm		
			Duration: 8h		
70	Yang et al. 2018	Total Cr/metals measured	Sampling: P	Prep: Each of the PVC membrane was conditioned before and after air sampling at a temperature of 21 ± 2°C and a relative humidity of 50 ± 3% for 48h. Then, the membrane was weighted twice using a balance with a 0.01mg resolution (Mettler-Toledo MX5 scale, Mettler-Toledo, Columbus, OH, USA).	No Cr(VI) speciation (total Cr/total metals only); LOD/LOQ not reported
			Fraction: Inhalable	Analytical method: NIOSH analytical method 7301	
			Thoracic	Instrument/notes: Inductively coupled plasma mass spectrometry (Agilent 7500ce ICP-MS (WA, USA))	
			Respirable	To divide welding fume into WS and WI, the Ghio method19) was followed. Each PVC membrane was placed into a 50-ml polypropylene tube with 40 ml of deionized water and agitated for 96h. The membrane was removed, and the aqueous extract was centrifuged at 2,500 × g for 30min, with the supernatant collected. This procedure was performed twice for each membrane. The two parts of the supernatant were pooled and then filtrated using a syringe filter holder (SWINNEX, Millipore, Billerica, MA, USA), cooperated with a mixed-cellulose ester (MCE) filter. Finally, the PVC membrane and the MCE filter were separately digested for analysis of WI metals.	
			Different fraction:	And the pooled supernatant was for analysis of WS metals by following the NIOSH analytical method 7301 with two modifications: (i) the hotplate was replaced with a microwave digester (CEM Corp., NC, USA), and (ii) the digesting acid was replaced with ultra-pure nitric acid (Sigma-Aldrich Inc., St. Louis, USA).	
			<0.52 μm (assuming the lower limit: 0.1 μm)		
			0.52–0.93 μm		
			0.93–1.55 μm		
			1.55–3.50 μm		

			3.50–6.00 µm		
			6.00–9.80 µm		
			9.80–14.80 µm		
			14.80–21.30 µm		
			>21.30 µm (assuming the upper limit: 31.35 µm)		
			Filter/substrate: 34-mm perforated Polyvinyl chloride (PVC) membrane (0.8 µm pore size; SKC Corp)		
			Flow: 2 l/min		
			Duration: 2h		
71	Lee et al. 2023	Total Cr/metals measured	Sampling: Stat.	Prep: Before and after the sampling, filters were quantified according to NMAMs 0500 & 0600 and weighed using an electronic scale (Mettler Toledo, AB204-S, Switzerland) with an accuracy up to 1 µg.	No Cr(VI) speciation (total Cr/total metals only); LOD/LOQ not reported
			Fraction: TSP	The polyvinyl chloride filters after weighing for a gravimetric analysis were analyzed according to the NIOSH NMAM 7304 method.	
			Respirable	Analytical method: Filter quantification: NMAMs 0500 & 0600	
			Method/sampler: Gravimetric analysis - TSP: NIOSH NMAM 0500	Gravimemtric: NIOSH NMAM 7304 method	
			Filter/substrate: Polyvinyl chloride filters (37 mm, pore size 5 µm, SKC, USA); support pad	Instrument/notes: Cr: Inductively coupled plasma mass spectrometer (Optima 3000, Perkin-Elmer, USA).	
			Flow: TSP: 2 l/min	Each filter was put into a polytetrafluoroethylene vessel and 5 mL of 70% nitric acid was injected for acidic digestion using a Mars System (CEM, model no. 910900, Matthews. NC, USA). Then, the suspension was diluted to a volume of 35 mL with distilled water. For each sample, a 1 mL solution was diluted to a total volume of 10 mL at a ratio of 1:9 with 5% distilled water.	
			Respirable: 2.5 l/min	(Real-time monitoring of airborne particles: optical particle sizer (OPS, Model 3330, TSI Inc., USA) for a size range of 0.3–10 µm. A scanning mobility particle sizer (SMPS, Model 3910, TSI Inc., USA), which can assess a size range of 10–420 nm, was used to evaluate the number concentration of the fine airborne particles, including the nano materials in the air at each site).	
			Duration: 3h		
72	Hanser et al. 2022	Total Cr/metals measured	Sampling: P	Analytical method: Air samples: INRS Metropol M−125 method (INRS, 2016)	No Cr(VI) speciation (total Cr/total metals only); Sampling approach/parameters incompletely reported
			Stat.	Air samples: ISO 15202–2 (ISO, 2001)	
			Fraction: Inhalable	Instrument/notes: Urine samples: inductively coupled plasma mass spectrometry (NexION 350X, Perkin Elmer, USA), on a system equipped with a collision/reaction cell (CRC). Urine samples were diluted 10-fold with a solution containing 1% (v/v)	

				HNO3 and 0.01% (v/v) Triton X-100.	
			Filter/substrate: Air: 37-mm PVC capsule fused to a cellulose acetate filter (Accu-CapTM, SC, USA)	Urine samples: Creatinine concentrations were determined in urinary samples by the Jaffe colorimetric method (Jaffé, 1886), using a Daytona-type analyzer (Randox, Antrim, UK).	
			Flow: Air: 2 l/min	Air samples: Plasma emission spectrometry (ICP-OES 5100, Agilent Technologies, USA) according to the INRS Metropol M−125 method (INRS, 2016) and ISO 15202–2 (ISO, 2001). The PVC capsule was rinsed with approximately 10 mL of demineralized water. The cellulose acetate filter was mineralized by applying 2 mL of reverse aqua regia and heating for 30 min at 150 °C. The product of this treatment was diluted to 25 mL with distilled water and filtered through a 0.45-μm hydrophilic Teflon filter.	
			Duration: Air: 8h	LOD: Not reported; LOQ: Air	
				LOQ: 0.03 ug/m3	
73	Sakebayeva et al. 2018	Total Cr/metals measured (inferred)	Sampling: Not reported	Not reported	No Cr(VI) speciation (total Cr/total metals only); Sampling approach/parameters incompletely reported; Flow rate not consistently reported; Sampling duration not consistently reported
			Fraction: Not reported		
74	Gravel et al. 2023	Total Cr/metals measured	Sampling: P	Analytical method: Air samples: IRSST method MA-362	No Cr(VI) speciation (total Cr/total metals only); Sampling approach/parameters incompletely reported; Protocol/standard not explicitly referenced in the paper
			Fraction: Not reported	Wipe: IRSST method MA-362	
			Method/sampler: Wipe: IRSST method I-MAT-016	Urine: IRSST method MA-361	
			Filter/substrate: Air: Pre-weighed 37 mm MCE filters (0.8 μm pores)	Instrument/notes: Air samples: inductively-coupled plasma-mass spectrometry (ICP/MS)	
			Flow: Air: 2 l/min	Wipe: inductively-coupled plasma-mass spectrometry (ICP/MS)	
			Duration: Air: The average sampling duration was 448 min (7.5 h)	Urine: Cr, Co and Ni were analyzed by ICP/MS (IRSST method MA-361). Urine specific gravity and creatinine were analyzed in the respective laboratories for each analysis.	
				LOD: Not reported; LOQ: Wipe sampling: 0.2 ug/sample	
75	Newton et al. 2021	Total Cr/metals measured	Sampling: P	Prep: Personal: Filters and their cassettes were pre- and post-weighed in a temperature and humidity-controlled weighing room at Johns Hopkins School of Public Health using a Mettler Toledo microbalance (Columbus, OH; ± 0.001 mg).	No Cr(VI) speciation (total Cr/total metals only); Sampling approach/parameters incompletely reported; Protocol/standard not explicitly referenced in the paper
			Stat.	LOD: Nine metals were assessed in the diluted digests from the IOM samples: vanadium, chromium, man-	

				ganese, cobalt, copper, nickel, zinc, molybdenum, and lead Eight IOM filters were below the method LOD for lead (7 ng/filter), and LOD/ √ 2 p was imputed for those exposures.; LOQ: Not reported	
			Fraction: PM10		
			Inhalable		
			Filter/substrate: IOM samplers, cassettes, and mixed cellulose ester (MCE) filters (25 mm, 0.8 mm) were purchased from SKC Inc. (Eighty Four, PA).		
			Flow: *Area - XRF: 16.7 LPM		
			*Area - IOM: 2 LPM		
			Duration: *Area - XRF: The EDXRF ran continuously for 1 week in each of the three shops		
			*Personal: 7AM to 6 PM		
76	Park et al. 2017	Total Cr/metals measured	Sampling: P	Instrument/notes: *Field portable X-ray fluorescence (FP-XRF): the particle-laden substrates were subjected to microwave-assisted acid digestion. The digestion protocol was a slightly modified version of the protocol used to dissolve TiO2 nanoparticles in our previous study (Mudunkotuwa et al. 2016). Briefly, the particle laden substrates were carefully placed in Teflon digestion vessels, a combination of conc. H2SO4 (6 mL) and conc. HNO3 (3 mL) was added, and the substrates were digested using a microwave digestion system (MARS 6, CEM Corporation, Matthews, NC, USA) for 45 min. The digestate was added to vials containing water (10 mL) and allowed to degas overnight in the fume hood. An additional dilution step (10 times dilution) was carried out to reduce the acidity of the medium. Finally, the solutions were topped up to 30 mL with water and analyzed by ICP-MS. The Fe and Cr content was analyzed and the values for mass per substrate were blank subtracted.	No Cr(VI) speciation (total Cr/total metals only); Sampling approach/parameters incompletely reported
			Fraction: 10 nm - 20 µm	*ICP-MS	
			Filter/substrate: *In order to reduce interference from the substrate holders during FP-XRF metals analysis, custom polyether ether ketone (PEEK) substrate holders were made and polycarbonate (PC) substrates were used for collection with the nano-MOUDI. PC filters (PCT0247100, pore size of 0.2 µm, diameter of 47 mm, Sterlitech, Kent, WA, USA) were coated with silicone oil (Heavy-duty silicone spray, Part #07041, MSP Corp., Shoreview, MN, USA) to prevent particle bounce.	LOD: LOD - PC substrate: 0.14 ug	

			*A mixed cellulose ester (MCE) filter (FMCE847, pore size of 0.8 μm, diameter of 47 mm, Zefon International, Inc., Ocala, FL, USA) was used as a backup filter.	LOD - MCE filter: 0.21 ug; LOQ: Not reported	
			Flow: Nano MOUDI: 10 L/min		
			Duration: 4h		
77	Cate et al. 2014	Both (Cr(VI) + total Cr/metals)	Sampling: Stat.	Analytical method: Sample preparation: EPA Method 3050B	LOD/LOQ not reported
			Fraction: <10 μm	ICP-OES analysis: EPA Method 6010B	
			Filter/substrate: Mixed cellulose ester (MCE) filters were purchased from Fisher Scientific Company (Pittsburgh, PA, USA) - 37-mm MCE filters (0.8 μm pore size)	Instrument/notes: *Inductively coupled plasma-optical emission spectroscopy (ICP-OES)	
			Whatman No. 1 qualitative-grade filter paper was purchased from General Electric Company (Schenectady, NY, USA)	*Colorimetric μPADs	
			Flow: 4 l /min	Following sample collection, 10-mm punches were taken from each filter and subjected to microwave-assisted acid digestion. Wetting and extraction efficiency was enhanced by pipetting 20 μl of surfactant [sodium dodecyl sulfate (SDS), 5 mM] onto each punch followed by air drying prior to sample digestion. To digest the metals in the welding fume, 5 μl of concentrated HNO3/SDS (5mM) was added to the punch along with 30 μl deionized water. Each punch was placed in a microwave (1100W) for 15 s. A second water/SDS (5mM) mixture (30 μl) was again added to the punch (to keep the filter wet), followed by another 15 s in the microwave; this wetting/microwave step was repeated twice. After digestion, the filter punch was neutralized by adding 10 μl of sodium bicarbonate (0.5M, pH 9.5), dried, and placed on the sample zone of the μPAD. For each test, a poly(dimethylsiloxane) (PDMS) lid, designed to reduce eluent evaporation and to distribute pressure evenly across the paper surface, was placed on top of filter punch/μPAD. The lid also contained openings above the sample (3mm diameter) and detection (5mm diameter) zones for solvent/buffer addition. Acetate buffer (40 μl, 0.1M, pH 4.5) was next added to the sample zone and a 300g weight was placed on the PDMS lid to help stabilize flow across the device. Metal detection was accomplished in ~20min after the eluent had completely dried. Devices were then analyzed using a common flatbed scanner.	
			Duration: 8h	For quantitation, devices were scanned using a desktop flatbed scanner (XEROX DocuMate 3220), providing a high resolution, well-focused image.	
78	López et al. 2023	Total Cr/metals measured	Sampling: Stat.	Prep: Aerosol sampling instrumentation: gravimetric mass determination.	No Cr(VI) speciation (total Cr/total metals only); Flow

					rate not consistently reported; LOD/LOQ not reported; Protocol/standard not explicitly referenced in the paper
			Fraction: 15 nm – 10 µm	Instrument/notes: Aerosol sampling instrumentation: acid digestion of the substrates (Querol et al., 2001) and determination of major and trace elements by Inductively Coupled Plasma Mass Spectrometry ICP-MS and Atomic Emission Spectroscopy Inductively Coupled Plasma ICP-AS.	
			Filter/substrate: Aerosol sampling instrumentation: pre-weighed polycarbonate substrates (25 mm in diameter)		
			Duration: Aerosol sampling instrumentation: 6h		
79	Vattanasit et al. 2021	Total Cr/metals measured	Sampling: Stat.	Analytical method: Hand Dust Wipe: NIOSH method 9102	No Cr(VI) speciation (total Cr/total metals only)
			Fraction: Respirable	Instrument/notes: Ambient air:. Ashing acid (4:1 v/v of HNO3 and HClO4) was used to extract the metals on a hotplate at an internal temperature of 150°C until the solution became clear. The extract was analyzed for the metal concentrations by inductively coupled plasma-optical emission spectrometry (ICP-OES, Perkin Elmer model Avio 200, USA).	
			Method/sampler: Ambient air: modified NIOSH method 7300	Hand Dust Wipe: The wipe samples were extracted for the metals according to NIOSH method 9102 [19]. Briefly, the wipe samples or blank wipes were extracted using 20ml of concentrated HNO3 and 1ml of concentrated HClO4. The reaction was run for 30min at room temperature and 2.5h on a hotplate at an internal temperature of 150°C until the solution became clear. The extract was analyzed for the metal concentrations by ICP-OES (Perkin Elmer model Avio 200, USA).	
			Filter/substrate: Ambient air: Polyvinyl chloride membrane filters (37mm) with a pore size of 5µm	LOD: LOD (ppb)	
			Flow: Ambient air: 1.5 L/min	Respirable dust: 1.8 ppb	
			Duration: Ambient air: 8h	Hand dust wipe: 0.9 ppb; LOQ: LOQ (ppb)	
			Hand Dust Wipe: end of the 8h work shift	Respirable dust: 6 ppb	
				Hand dust wipe: 3 ppb	
80	Wang et al. 2017	Cr(VI) measured	Sampling: Stat.	Prep: The sampled filters, once weighed, were cut into halves for different extraction and analysis protocols. One half was used for Cr6+ analysis, while the other half was used for other inorganic oxides analysis.	Sampling approach/parameters incompletely reported; Sampling duration not consistently reported
			Fraction: Inhalable	Analytical method: Cr6+ analysis: modified NIOSH Method 7604 (NIOSH, 1994).	
			Filter/substrate: Glass fiber filters (Whatman GF/A-90, Maidstone, Kent, UK)	Instrument/notes: *Fume gravimetrical measurement: analytical balance	
			Flow: 0.5 m3/min.	*Cr(VI) in the form of chromate: IC. The Cr6+ analysis in this study fol-	

				lowed a modified NIOSH Method 7604 (NIOSH, 1994). The half filter was heat-assisted extracted using 10 ml of a solution of 2% sodium hydroxide (NaOH) and 3% sodium carbonate (Na2CO3). The extracts were then analyzed using a conductivity-based ion chromatograph (IC; Dionex ICS-1600, Sunnyvale, CA, USA) equipped with an anion analytical column (Dionex AS4A, Sunnyvale, CA, USA) in the form of chromate (CrO42−). The mass conversion of chromate to Cr6+ was done by multiplying a factor of 0.45 (the ratio of molecular weights of Cr6+ and CrO42−).	
				*Inorganic oxides in the form of acids (PO4; SO4; NO2; NO3): IC	
				*Particle size distribution (fine fraction): Butanol-based scanning mobility particle sizer - SMPS (SMPS; TSI 3936, Shoreview, MN, USA)	
				*Particle size distribution (coarse fraction): Aerodynamic particle sizer - APS (APS; TSI 3314, Shoreview, MN, USA)	
				*Particle morphology: transmission electron microscope (TEM)	
				LOD: LOD	
				Fume gravimetrical measurement: 0.1 mg/filter	
				Cr(VI) in the form of chromate: 6.7 ug/sample	
				Inorganic oxides in the form of acids (PO4; SO4; NO2; NO3): 1.0 ug/sample	
				Fine particles: 10^7 #/cm3 (upper); 10^3#/cm3 (lower)	
				Coarse particles: 10^5 #/cm3 (upper); 10^-2#/cm3 (lower)	
				Particle morphology: 10 nm; LOQ: Not reported	
81	Mitra and Das 2022	Total Cr/metals measured	Sampling: Stat.	Prep: After sampling, filters were once again weighed to gravimetrically determine PM2.5 concentrations	No Cr(VI) speciation (total Cr/total metals only); LOD/LOQ not reported; Protocol/standard not explicitly referenced in the paper
			Fraction: PM2.5	Instrument/notes: SEM: Scanning electron microscopy (SEM) coupled with energy dispersive X-ray analysis (EDS) were performed for selected filters on a JEOL JSM-7600F (Field Emission Scanning Electron Microscope) to determine the physical characteristics and information regarding the chemical composition of the airborne particles.	
			Filter/substrate: Pre-weighted and pre-cleaned 47mm Teflon filters	The trace metal concentrations of the samples (bio-available fraction and residual fraction) and the blanks were determined using a sector-field inductively coupled plasma mass spectrometer (SF-ICP-MS, an Element 2 from Thermo Fisher Scientific). A multi-element standard solution (IV-ICPMS-71A from Inorganic Ventures) diluted to appropriate concentrations depending on the signal range was used to measure the metal concentrations.	
			Flow: 10L/min	SRM 2783 of Urban Particulate Matter samples were used to validate the	

				method.	
			Duration: 8h (10 AM - 6PM)		
83	Jafari et al. 2023	Both (Cr(VI) + total Cr/metals)	Sampling: P	Analytical method: OSHA ID-121 (Method & ID-121, 2002)	Sampling approach/parameters incompletely reported
			Fraction: Not reported	Instrument/notes: Air filters were digested with the appropriate acid solutions in order to extract metals.	
			Method/sampler: OSHA ID-121 (Method & ID-121, 2002)	A graphite furnace atomic absorption spectrometer (Perkin Elmer 5100 AAS) was used to measure the concentration of Cd, Cr, Pb, and As in air samples.	
			Filter/substrate: 37-mm cellulose ester membrane filter (0.8-micron pore size)	LOD: LOD: 0.16 ug/m3; LOQ: Not reported	
			Flow: 2 L/min		
			Duration: 4h		
84	Peters et al. 2015	Cr-related (secondary/estimated)	Sampling: Not reported	Not reported	Cr species unclear/secondary estimate (interpret with caution); Sampling approach/parameters incompletely reported; Flow rate not consistently reported; Sampling duration not consistently reported
			Fraction: Not reported		
85	Pesch et al. 2015	Cr(VI) measured	Sampling: P	Instrument/notes: Cr(VI) concentrations were determined spectrophotometrically at 540 nm after alkaline extraction from the filter and following a reaction with diphenylcarbazide (DPC) in a strongly acidic solution (Hagemann and Hahn, 1989).	LOD/LOQ not reported
			Fraction: Inhalable		
			Method/sampler: EN 481, 1993		
			Filter/substrate: Quartz-fibre filters		
			Flow: 3.5 L/min		
			Duration: Median duration of sampling was 2 h		
86	Pourhassan et al. 2023	Total Cr/metals measured	Sampling: P	Analytical method: NIOSH 7300 method	No Cr(VI) speciation (total Cr/total metals only); Sampling approach/parameters incompletely reported
			Fraction: Not reported	Instrument/notes: Inductively coupled plasma mass spectroscopy (ICP-MS) (Agilent 7500a, Agilent Co. USA) was used for sample analyzing.	
			Method/sampler: NIOSH 7300	Each MCE filter was cut into separate pieces and placed in a 100-ml beaker containing 5 ml nitric acid (HNO3) and then added one drop of Hydrofluoric acid (HF). Then, the solution was covered and placed on a hot plate at the temperature of 220 °C for 2.5 h. Finally, 5 ml of hydrochloric acid (HCL) was added to the beaker and the solution was transferred to a 10-ml plastic bottle. The digested filters were diluted to 10 mL using deionized distilled water. The metals' concentrations were read on a standard curve prepared from a standard solution of 1 mg/mL concentration (Trace Metal Analysis Standard for AAS/ICP, Baker Instra-Analysed. 69–70%) [18].	

			Filter/substrate: Mixed Cellulose Membrane (MCE) Filter (25 mm, 0.8 µm, SKC Inc., USA)	LOD: Not reported; LOQ: LOQ: 0.5 µg/m3	
			Flow: 2 L/min.		
			Duration: 4h		
87	van Ree et al. 2023	Both (Cr(VI) + total Cr/metals)	Sampling: P	Prep: Gravimetric analysis post-measurement in order to determine the mass concentration of the powder to which AM operators are exposed. The gravimetric analysis determined the mass of particles on each sample was conducted based on MDHS Method 14, National Institute for Occupational Safety and Health (NIOSH) Method 0500, 0600, and GME 16/2/3/2/3 by the laboratory.	Sampling duration not consistently reported; LOD/LOQ not reported
			Fraction: Inhalable	Analytical method: Gravimetric analysis:	
			Method/sampler: MDHS 14/4	MDHS Method 14, National Institute for Occupational Safety and Health (NIOSH) Method 0500, 0600	
			Filter/substrate: *IOM: foam insert and a 25 mm mixed cellulose ester (MCE) filter	GME 16/2/3/2/3	
			Flow: *IOM: 2 L per minute (L/min)	ICP-OES: NIOSH Method 7303	
				Cr(VI): NIOSH Method 7600	
				Instrument/notes: *Inductively coupled plasma optical emission spectroscopy (ICP-OES).	
				*Scanning electron microscopy (SEM): The SEM was conducted using the Phenom pro-desktop Scanning Electron Microscope (Phenom PRO Desktop SEM, Phenom-World B., Eindhoven, The Netherlands). Adhesive carbon strips were used to collect powder for analysis; the strip was placed inside the instrument and observed at 10 kV magnification to visualize particles.	
				*Particle size distribution (PSD): PSD analysis was conducted using the Malvern Morphologi G3 system (Malvern Panalytical Ltd., Malvern, UK). About 5 mm3 of the sample was dispersed onto the sample plate by the instrument's dispersion unit.	
				*Particle counters were placed as close as possible to the machine, within 1.5 m, to capture the particle number concentration (p/cm3) over time in the area. Particle counters included: TSI P-Trak® Ultrafine particle counter model 8525 (TSI Inc., Shoreview, MN, USA) and the Grimm Portable Laser Aerosol Spectrometer model 11-A (GRIMM Aerosol Technik GmbH & Co., Muldestausee, Germany). The particle size range of the instruments differs as follows: the P-Trak® detects the smallest range of particles from 0.02 to 1 µm while the Grimm has 31 channels, detecting particles from 0.25 to 32 µm.	

References

Arsal Yıldırım S, Pekey B, Pekey H. (2020) Assessment of occupational exposure to fine particulate matter in dental prosthesis laboratories in Kocaeli, Turkey. *Environ Monit Assess*; **192**: 667.

Bau S, Rousset D, Payet R, Keller F-X. (2020) Characterizing particle emissions from a direct energy deposition additive manufacturing process and associated occupational exposure to airborne particles. *J Occup Environ Hyg*; **17**: 59–72.

Bennett JS, Marlow DA, Nourian F, Breay J, Hammond D. (2016) Hexavalent chromium and isocyanate exposures during military aircraft painting under crossflow ventilation. *J Occup Environ Hyg*; **13**: 356–71.

Berlinger B, Harper M. (2018) Interlaboratory comparison for the determination of the soluble fraction of metals in welding fume samples. *J Occup Environ Hyg*; **15**: 152–6.

Cate DM, Nanthasurasak P, Riwkulkajorn P, L'Orange C, Henry CS, Volckens J. (2014) Rapid detection of transition metals in welding fumes using paper-based analytical devices. *Ann Occup Hyg*; **58**: 413–23.

Cena LG, Chisholm WP, Keane MJ, Chen BT. (2015) A Field Study on the Respiratory Deposition of the Nano-Sized Fraction of Mild and Stainless Steel Welding Fume Metals. *J Occup Environ Hyg*; **12**: 721–8.

Cena LG, Keane MJ, Chisholm WP, Stone S, Harper M, Chen BT. (2014) A novel method for assessing respiratory deposition of welding fume nanoparticles. *J Occup Environ Hyg*; **11**: 771–80.

Chen Y-C, Coble JB, Deziel NC, et al. (2014) Reliability and validity of expert assessment based on airborne and urinary measures of nickel and chromium exposure in the electroplating industry. *J Expo Sci Environ Epidemiol*; **24**: 622–8.

Decharat S. (2015) Chromium Exposure and Hygienic Behaviors in Printing Workers in Southern Thailand. *J Toxicol*; **2015**: 607435.

Dueck ME, Rafiee A, Mino J, et al. (2021) Welding Fume Exposure and Health Risk Assessment in a Cohort of Apprentice Welders. *Ann Work Expo Health*; **65**: 775–88..

Engelsman M, Snoek MF, Banks APW, et al. (2019) Exposure to metals and semivolatile organic compounds in Australian fire stations. *Environ Res*; **179**: 108745.

Eriksen Hammer S, Halvorsen JØ, Graff P, Ervik TK. (2022) Characterisation of Particles Emitted during Laser Cutting of Various Metal Sheets and an Exposure Assessment for the Laser Operators. *Int J Environ Res Public Health*; **19**.

Galarneau J-M. (2022) Construction and Calibration of an Exposure Matrix for the Welding Trades. *Ann Work Expo Health*; **66**: 178–91.

Galvão MF de O, Cabral T de M, de André PA, et al. (2014) Cashew nut roasting: chemical characterization of particulate matter and genotocixity analysis. *Environ Res*; **131**: 145–52.

Gerding J, Peters C, Wegscheider W, et al. (2021) Metal exposure of workers during recycling of electronic waste: a cross-sectional study in sheltered workshops in Germany. *Int Arch Occup Environ Health*; **94**: 935–44.

Ghobakhloo S, Mostafaii GR, Khoshakhlagh AH, Moda HM, Gruszecka-Kosowska A. (2024) Health risk assessment of heavy metals in exposed workers of municipal waste recycling facility in Iran. *Chemosphere*; **346**: 140627.

Graff P, Ståhlbom B, Nordenberg E, Graichen A, Johansson P, Karlsson H. (2017) Evaluating Measuring Techniques for Occupational Exposure during Additive Manufacturing of Metals: A Pilot Study. *J Ind Ecol*; **21**: S120–9.

Gravel S, Roberge B, Calosso M, Gagné S, Lavoie J, Labrèche F. (2023) Occupational health and safety, metal exposures and multi-exposures health risk in Canadian electronic waste recycling facilities. *Waste Manag*; **165**: 140–9.

Gu Y, Xiang D, Cai K, et al. (2023) Ultrasensitive Electrochemical Detection of Cr(VI) in the Air of Workplace Using the Bismuth Film Modified Electrode. *Electrocatalysis*; **14**: 78–87.

Hamid A, Riaz A, Noor F, Mazhar I. (2023) Assessment and mapping of total suspended particulate and soil quality around brick kilns and occupational health issues among brick kilns workers in Pakistan. *Environ Sci Pollut Res Int*; **30**: 3335–50.

Hamzah NA, Mohd Tamrin SB, Ismail NH. (2016) Metal dust exposure and lung function deterioration among steel workers: an exposure-response relationship. *Int J Occup Environ Health*; **22**: 224–32.

Hanser O, Melczer M, Martin Remy A, Ndaw S. (2022) Occupational exposure to metals among battery recyclers in France: Biomonitoring and external dose measurements. *Waste Manag*; **150**: 122–30.

Huang H, Li H, Li X. (2016) Physicochemical Characteristics of Dust Particles in HVOF Spraying and Occupational Hazards: Case Study in a Chinese Company. *Journal of Thermal Spray Technology*; **25**: 971–81.

Jafari A, Asadyari S, Moutab Sahihazar Z, Hajaghazadeh M. (2023) Monte Carlo-based probabilistic risk assessment for cement workers exposed to heavy metals in cement dust. *Environ Geochem Health*; **45**: 5961–79.

Järvelä M, Huvinen M, Viitanen A-K, et al. (2016) Characterization of particle exposure in ferrochromium and stainless steel production. *J Occup Environ Hyg*; **13**: 558–68.

Jiang Z, Schenk L, Assarsson E, et al. (2024) Hexavalent chromium still a concern in Sweden - Evidence from a cross-sectional study within the SafeChrom project. *Int J Hyg Environ Health*; **256**: 114298.

Julander A, Lundgren L, Skare L, et al. (2014) Formal recycling of e-waste leads to increased exposure to toxic metals: an occupational exposure study from Sweden. *Environ Int*; **73**: 243–51.

Kalteh S, Mozaffari S, Molaei I, Maleki R. (2020) Health risk assessment of metal fumes in an Iranian Mineral Salt company. *Journal of Air Pollution and Health*; **5**.

Kamaludin NH, Jalaludin J, Mohd Tamrin SB, Md Akim A, Martiana T, Widajati N. (2020) Exposure to Silica, Arsenic, and Chromium (VI) in Cement Workers: A Probability Health Risk Assessment. *Aerosol Air Qual Res*; **20**: 2347–70..

Kato N, Yamada M, Ojima J, Takaya M. (2022) Analytical method using SEM-EDS for metal elements present in particulate matter generated from stainless steel flux-cored arc welding process. *J Hazard Mater*; **424**: 127412.

Kettelarij J, Nilsson S, Midander K, Lidén C, Julander A. (2016) Snapshot of cobalt, chromium and nickel exposure in dental technicians. *Contact Dermatitis*; **75**: 370–6.

Keyter M, Van Der Merwe A, Franken A. (2019) Particle size and metal composition of gouging and lancing fumes. *J Occup Environ Hyg*; **16**: 643–55.

Khalili M, Nasrabadi T. (2023) Assessment of occupational health risk due to inhalation of chemical compounds in an aircraft maintenance, repair, and overhaul company. *Environ Sci Pollut Res Int*; **30**: 57558–70.

Khoshakhlagh AH, Yazdanirad S, Saberi HR, Liao P-C. (2023) Health risk assessment of exposure to various vapors and fumes in a factory of automobile manufacturing. *Heliyon*; **9**: e18583.

Laitinen J, Koponen H, Sippula O, et al. (2017) Peak exposures to main components of ash and gaseous diesel exhausts in closed and open ash loading stations at biomass-fuelled power plants. *Chemosphere*; **185**: 183–91.

Lau WKY, Liang P, Man YB, Chung SS, Wong MH. (2014) Human health risk assessment based on trace metals in suspended air particulates, surface dust, and floor dust from e-waste recycling workshops in Hong Kong, China. *Environ Sci Pollut Res Int*; **21**: 3813–25.

Lee M, Jung S, Do G, Yang Y, Kim J, Yoon C. (2023) Measurement of Airborne Particles and Volatile Organic Compounds Produced During the Heat Treatment Process in Manufacturing Welding Materials. *Saf Health Work*; **14**: 215–21.

Lehnert M, Goebel A, Zschiesche W, et al. (2022) How to Reduce the Exposure of Welders to an Acceptable Level: Results of the InterWeld Study. *Ann Work Expo Health*; **66**: 192–202.

Lehnert M, Weiss T, Pesch B, et al. (2014) Reduction in welding fume and metal exposure of stainless steel welders: an example from the WELDOX study. *Int Arch Occup Environ Health*; **87**: 483–92.

López M, López-Lilao A, Romero F, et al. (2023) Size-resolved chemical composition and toxicity of particles released from refit operations in shipyards. *Sci Total Environ*; **880**: 163072.

Mariem Nafti Radhouane Chakroun CHBH, Nouaïgui H. (2017) Determination of Chromium (VI) in Airborne Particulate Matter by Electrothermal Atomic Absorption Spectrometry. *Anal Lett*; **50**: 2012–22.

Martin J, Bello D, Bunker K, et al. (2015) Occupational exposure to nanoparticles at commercial photocopy centers. *J Hazard Mater*; **298**: 351–60.

Mehrifar Y, Zeverdegani SK, Rismanchian M. (2020) Chemical pollutants in the respiratory zone of welders: Determination of concentrations and hazard analysis. *Work*; **67**: 591–8.

Miettinen M, Torvela T, Leskinen JTT. (2016) Physicochemical Characterization of Aerosol Generated in the Gas Tungsten Arc Welding of Stainless Steel. *Ann Occup Hyg*; **60**: 960–8.

Mitra S, Das R. (2022) Health risk assessment of construction workers from trace metals in PM(2.5) from Kolkata, India. *Arch Environ Occup Health*; **77**: 125–40..

Newton A, Adams K, Serdar B, Dickinson LM, Koehler K. (2021a) Personal and area exposure assessment at a stainless steel fabrication facility: an evaluation of inhalable, time-resolved PM(10,) and bioavailable airborne metals. *J Occup Environ Hyg*; **18**: 90–100.

Newton A, Rule AM, Serdar B, Koehler K. (2023) Laboratory comparison of field portable X-ray fluorescence spectrometer (FP-XRF) and inductively coupled plasma mass spectrometry (ICP-MS) for determination of airborne metals in stainless steel welding fume. *J Occup Environ Hyg*; **20**: 536–44.

Newton A, Serdar B, Adams K, Dickinson LM, Koehler K. (2021b) Lung deposition versus inhalable sampling to estimate body burden of welding fume exposure: A pilot sampler study in stainless steel welders. *J Aerosol Sci*; **153**: 105721.

Nurul AH, Shamsul BMT, Noor Hassim I. (2016) Assessment of dust exposure in a steel plant in the eastern coast of peninsular Malaysia. *Work*; **55**: 655–62.

Oddone E, Pernetti R, Fiorentino ML, et al. (2022) Particle measurements of metal additive manufacturing to assess working occupational exposures: a comparative analysis of selective laser melting, laser metal deposition and hybrid laser metal deposition. *Ind Health*; **60**: 371–86.

Oginawati K, Susetyo SH, Rosalyn FA, Kurniawan SB, Abdullah SRS. (2021) Risk analysis of inhaled hexavalent chromium (Cr(6+)) exposure on blacksmiths from industrial area. *Environ Sci Pollut Res Int*; **28**: 14000–8.

Ogundele LT, Owoade OK, Hopke PK, Olise FS. (2017) Heavy metals in industrially emitted particulate matter in Ile-Ife, Nigeria. *Environ Res*; **156**: 320–5.

Onat B, Çalışkan NS, Şahin ÜA, Uzun B. (2020) Assessment of the health risk related to exposure to ultrafine, fine, and total particulates and metals in a metal finishing plant. *Environ Sci Pollut Res Int*; **27**: 4058–66.

Park JH, Mudunkotuwa IA, Crawford KJ, Anthony TR, Grassian VH, Peters TM. (2017) Rapid Analysis of the Size Distribution of Metal-Containing Aerosol. *Aerosol Sci Technol*; **51**: 108–15.

Pesch B, Kendzia B, Hauptmann K, et al. (2015) Airborne exposure to inhalable hexavalent chromium in welders and other occupations: Estimates from the German MEGA database. *Int J Hyg Environ Health*; **218**: 500–6.

Pesch B, Lehnert M, Weiss T, et al. (2018) Exposure to hexavalent chromium in welders: Results of the WELDOX II field study. *Ann Work Expo Health*; **62**: 351–61.

Peters CE, Ge CB, Hall AL, Davies HW, Demers PA. (2015) CAREX Canada: an enhanced model for assessing occupational carcinogen exposure. *Occup Environ Med*; **72**: 64–71.

Pourbakhshi Y, Heidari M, Yahaei E, Ghiyasi S, Ebrahimi-Najafabadi H, Bozorgzadeh E. (2021) Dispersive Liquid–Liquid Microextraction Followed by Solidified Floating Organic Drop for Hexavalent Chromium Determination: a Method for Occupational and Environmental Exposure Monitoring for Heavy Metals. *Journal of Analytical Chemistry*; **76**: 714–20.

Pourhassan B, Beigzadeh Z, Nasirzadeh N, Karimi A. (2024) Application of Multiple Occupational Health Risk Assessment Models for Metal Fumes in Welding Process. *Biol Trace Elem Res*; **202**: 811–23.

van Ree M, du Preez S, du Plessis JL. (2023) Emissions and Exposures Associated with the Use of an Inconel Powder during Directed Energy Deposition Additive Manufacturing. *Int J Environ Res Public Health*; **20**.

Sakebayeva L, Karashova G, Kuspangaliyeva G, et al. (2018) Occupational health and safety risk assessment in chrome production. *Indian J Public Health Res Dev*; **9**: 423. p. 423.

Sarwar F, Alam K, Öztürk F, Koçak M, Malik RN. (2023) Appraising the characteristics of particulate matter from leather tanning micro-environments, their respirational risks, and dysfunctions amid exposed working cohorts. *Environ Monit Assess*; **195**: 1556.

Ščančar J, Berlinger B, Thomassen Y, Milačič R. (2015) Simultaneous speciation analysis of chromate, molybdate, tungstate and vanadate in welding fume alkaline extracts by HPLC-ICP-MS. *Talanta*; **142**: 164–9.

Sepahi Zoeram F, Ebrahimi AA, Mehrparvar AH, et al. (2022) Health risk assessment of inhalational exposure to heavy metals in drivers working in an urban desert city in the Middle East. *Environ Monit Assess*; **194**: 533.

Shaw L, Shaw D, Hardisty M, Britz-McKibbin P, Verma DK. (2020) Relationships between inhalable and total hexavalent chromium exposures in steel passivation, welding and electroplating operations of Ontario. *Int J Hyg Environ Health*; **230**: 113601.

Sikder AM, Hossain T, Khan MH, et al. (2017) Toxicity assessment of ash and dust from handmade gold jewelry manufacturing workshops in Bangladesh. *Environ Monit Assess*; **189**: 279.

Soltanpour Z, Rasoulzadeh Y, Ansarin K, Seyedrezazadeh E, Mohammadian Y. (2023) Carcinogenic and non-carcinogenic risk of exposure to metal fume in different types of welding processes. *Environ Sci Pollut Res Int*; **30**: 83728–34.

Spinazzè A, Spanu D, Della Bella P, et al. (2022) On the Determination of Cr(VI) in Cr(III)-Rich Particulates: From the Failure of Official Methods to the Development of an Alternative Protocol. *Int J Environ Res Public Health*; **19**.

Stanislawska M, Halatek T, Cieslak M, et al. (2017) Coarse, fine and ultrafine particles arising during welding - Analysis of occupational exposure. *Microchemical Journal*; **135**: 1–9. p. 1–9.

Vattanasit U, Sukchana J, Kongsanit S, Dumtip P, Sirimano V, Kongpran J. (2021) Toluene and Heavy Metals in Small Automotive Refinishing Shops and Personal Protection of the Workers in Nakhon Si Thammarat, Thailand. *J Environ Public Health*; **2021**: 8875666.

Viegas S, Martins C, Bocca B, et al. (2022) HBM4EU Chromates Study: Determinants of Exposure to Hexavalent Chromium in Plating, Welding and Other Occupational Settings. *International Journal of Environmental Research and Public Health 2022, Vol 19, Page 3683*; **19**: 3683.

Vincent R, Gillet M, Goutet P, et al. (2015) Occupational exposure to chrome VI compounds in French companies: results of a national campaign to measure exposure (2010-2013). *Ann Occup Hyg*; **59**: 41–51.

Wang J, Hoang T, Floyd EL, Regens JL. (2017) Characterization of Particulate Fume and Oxides Emission from Stainless Steel Plasma Cutting. *Ann Work Expo Health*; **61**: 311–20.

Wang Y-F, Kuo Y-C, Wang L-C. (2022) Long-term metal fume exposure assessment of workers in a shipbuilding factory. *Sci Rep*; **12**: 790.

Wu C-C, Liu H-M. (2014) Determinants of metals exposure to metalworking fluid among metalworkers in Taiwan. *Arch Environ Occup Health*; **69**: 131–8.

Wu C-M, Song CC, Chartier R, Kremer J, Naeher L, Adetona O. (2021) Characterization of occupational smoke exposure among wildland firefighters in the midwestern United States. *Environ Res*; **193**: 110541.

Yang S-Y, Lin J-M, Lin W-Y, Chang C-W. (2018a) Cancer risk assessment for occupational exposure to chromium and nickel in welding fumes from pipeline construction, pressure container manufacturing, and shipyard building in Taiwan. *J Occup Health*; **60**: 515–24.

Yang S-Y, Lin J-M, Young L-H, Chang C-W. (2018b) Mass-size distribution and concentration of metals from personal exposure to arc welding fume in pipeline construction: a case report. *Ind Health*; **56**: 356–63.

Zendehdel R, Fazli Z, Rezazadeh Azari M. (2019) Neurological risk assessment of co-exposure to heavy metals (chromium and nickel) in chromium-electroplating workers. *Work*; **63**: 355–60.
